# Geometric variation of the human tibia-fibula: a public dataset of tibia-fibula surface meshes and statistical shape model

**DOI:** 10.7717/peerj.14708

**Published:** 2023-02-16

**Authors:** Meghan Keast, Jason Bonacci, Aaron Fox

**Affiliations:** Centre for Sport Research, School of Exercise and Nutrition Sciences, Deakin University, Waurn Ponds, VIC, Australia

**Keywords:** Musculoskeletal model, Lower extremity, Statistical shape modelling, Bone model

## Abstract

**Background:**

Variation in tibia geometry is a risk factor for tibial stress fractures. Geometric variability in bones is often quantified using statistical shape modelling. Statistical shape models (SSM) offer a method to assess three-dimensional variation of structures and identify the source of variation. Although SSM have been used widely to assess long bones, there is limited open-source datasets of this kind. Overall, the creation of SSM can be an expensive process, that requires advanced skills. A publicly available tibia shape model would be beneficial as it enables researchers to improve skills. Further, it could benefit health, sport and medicine with the potential to assess geometries suitable for medical equipment, and aid in clinical diagnosis. This study aimed to: (i) quantify tibial geometry using a SSM; and (ii) provide the SSM and associated code as an open-source dataset.

**Methods:**

Lower limb computed tomography (CT) scans from the right tibia-fibula of 30 cadavers (male *n* = 20, female *n* = 10) were obtained from the New Mexico Decedent Image Database. Tibias were segmented and reconstructed into both cortical and trabecular sections. Fibulas were segmented as a singular surface. The segmented bones were used to develop three SSM of the: (i) tibia; (ii) tibia-fibula; and (iii) cortical-trabecular. Principal component analysis was applied to obtain the three SSM, with the principal components that explained 95% of geometric variation retained.

**Results:**

Overall size was the main source of variation in all three models accounting for 90.31%, 84.24% and 85.06%. Other sources of geometric variation in the tibia surface models included overall and midshaft thickness; prominence and size of the condyle plateau, tibial tuberosity, and anterior crest; and axial torsion of the tibial shaft. Further variations in the tibia-fibula model included midshaft thickness of the fibula; fibula head position relative to the tibia; tibia and fibula anterior-posterior curvature; fibula posterior curvature; tibia plateau rotation; and interosseous width. The main sources of variation in the cortical-trabecular model other than general size included variation in the medulla cavity diameter; cortical thickness; anterior-posterior shaft curvature; and the volume of trabecular bone in the proximal and distal ends of the bone.

**Conclusion:**

Variations that could increase the risk of tibial stress injury were observed, these included general tibial thickness, midshaft thickness, tibial length and medulla cavity diameter (indicative of cortical thickness). Further research is needed to better understand the effect of these tibial-fibula shape characteristics on tibial stress and injury risk. This SSM, the associated code, and three use examples for the SSM have been provided in an open-source dataset. The developed tibial surface models and statistical shape model will be made available for use at: https://simtk.org/projects/ssm_tibia.

## Introduction

Statistical shape models (SSM) offer a method to assess three-dimensional variation of structures, including the sources of variation in bones. Although SSM have been used widely to assess long bones (Zhang et al., 2014a; Nolte et al., 2020; Bruce et al., 2021) there are limited open-source datasets of this kind. SSM that use medical imaging can be costly and the funding or facilities needed to produce high quality imagery can be inaccessible. Segmentation of medical images and bone surface reconstruction requires specialized software, skill, and extensive time. Further, transforming the segmented data into three-dimensional models requires an intermediate level of computer coding knowledge. Attainment of coding proficiency is a steep learning curve that requires extensive mentorship. Overall, the creation of SSM can be an expensive and time costly process that requires advanced skills. A publicly available tibial shape model would be beneficial as it enables researchers who may be affected by the aforementioned hurdles to improve skills and adapt the code to meet their needs. Openly sharing code, data and instructional articles is beneficial to all fields of research (Pisani et al., 2016; Pronk, 2019). SSM can also further research in health, sport, and medicine with the potential to create a sample of computer simulated tibias, assess geometries suitable for medical equipment, and aid in clinical diagnosis.

The ability to characterize and understand bone shape and geometric variation is also an important aspect in skeletal research. Bone stress injuries caused by exercise are one area that may benefit from the increased understanding of bone shape and individual variation. Individuals who engage in high volumes of running, through recreational running or sport participation, are at an elevated risk for lower limb bone stress injuries (Rizzone et al., 2017). Tibial stress reactions and tibial stress fractures are the fifth and ninth most common running-related injuries, respectively (Taunton, 2002). Tibial stress injuries result, in part, from the mechanical fatigue of bone after cyclic loading (Burr et al., 1991). Tibial stress injuries are multifactorial in nature and can be caused by several intrinsic and extrinsic factors, one of these being skeletal geometry (Popp et al., 2019). Variations in tibial geometry such as smaller tibias (Crossley et al., 1999; Beck et al., 2014) and a thinner mid diaphysis (Popp et al., 2019) have been cited as a risk factor for tibial stress injury. Quantifying tibial anatomy using SSM and providing this as an open resource can assist in progressing our understanding of risk factors for tibial stress injury, as well as identify relevant interventions to reduce the risk of these injuries in running populations. Therefore, the aims of this article are: (i) to quantify tibial geometry using a SSM; and (ii) provide the SSM and associated code as an open-source dataset. This article and associated code will also demonstrate potential applications for the SSM to guide potential use.

## Methods

Lower limb computed tomography (CT) scans from the right tibia and fibula of 30 cadavers (male *n* = 20, female *n* =10) were obtained from the New Mexico Decedent Image Database (Edgar et al., 2020). The images included were from individuals with a mean (±standard deviation) age of 28.7 ± 6.7, living weight of 70.22 ± 11.36 kg, and living height of 176.06 ± 11.61 cm. Individuals whose records indicated participation in impact-based physical activities throughout life were selected for inclusion (*i.e*., team sports, dancing, recreational running and walking). This criterion was included in an effort to ensure participants were sampled from a generally active population. Participants were excluded if there was any noticeable damage to the right tibia-fibula or the inclusion of medical devices (*e.g*., plates, rods, or screws). The tibia and fibulas were segmented from the CT images using Mimics innovation suite (Materialise, Leuven, Belgium). Tibias were segmented into two surfaces representing the outer boundaries of the trabecular and cortical bone, while fibulas were segmented as one surface representing the outer shape of the entire bone. The cortical bone was segmented using a predefined threshold (lower bound 700 HU, upper bound 2,500 HU), all surfaces were checked, and manual corrections applied where necessary. The inner border of the cortical bone was used to specify the beginning of the trabecular bone, all area inside this was segmented as the trabecular bone, including the medulla cavity. The resolution of the CT scans used in this study was not high enough to allow intricate segmentation of the lattice structure of trabecular bone. The medulla cavity was included in the trabecular bone segmentation as this enabled us to describe changes in size and shape of the entire trabecular surface and medulla cavity without needing to create a cross section of the tibia. Landmarks on the lateral and medial malleoli, lateral and medial tibial condyle, fibula head, tibial tuberosity, anterior aspect of the tibia at 25%, 50% and 75% of the distance between the medial condyle and malleolus, and lateral fibula diaphysis 25% proximally from the malleoli were registered from the medical images on the segmented surfaces (Bruce et al., 2021).

The segmented bones (cortical and trabecular tibia; and fibula) were used to develop three SSM of the: (i) outer cortical surface of the tibia; (ii) combined outer cortical surface of the tibia and fibula; and (iii) combined model of the cortical and trabecular surfaces of the tibia. Throughout the remainder of this study these models will be referred to as the tibia model, tibia-fibula model and the cortical-trabecular model. The procedures used to create the SSM were applied identically across all three models. Prior to development of the SSM, the bones were aligned to the tibial reference system as per International Society of Biomechanics recommendations (Wu et al., 2002). This was done to simplify later steps in the surface registration processes. Surfaces were remeshed to have a matching number of points (tibia *n* = 3,500; fibula *n* = 1,500; trabecular *n* = 1,500). Nodal correspondence and registration were performed in MATLAB (R2019b; Mathworks, Natick, MA, USA). All surfaces were registered against the same target mesh (Bruce et al., 2021). Initially, all surfaces were meshed to a randomly selected target mesh in order to create a mean surface mesh. The surfaces were then re-registered to a new target mesh. The new target mesh was selected by comparing the average point to point error between the mean and sample surface, this target mesh was the surface with the lowest average point error. Nodal correspondence was performed using the coherent point drift algorithm (Myronenko & Song, 2010). This algorithm non-rigidly registers each surface mesh to the target mesh so that corresponding points between surfaces can be identified. The surface meshes were then rigidly aligned using the same coherent point drift algorithm with settings changed to perform a rigid registration with no scaling applied (Myronenko & Song, 2010).

Principal component analysis (PCA) was applied to the surface sets to obtain the three SSM. The principal components (PCs) that accounted for >95% of geometric variation in each SSM were retained (Smoger et al., 2017). Mean and peak error in surface node position, and the Jaccard index (*i.e*., a measure of volumetric similarity where values range from 0 to 1 indicating no to perfect similarity, respectively) (Real & Vargas, 1996) (mean ± 95% confidence intervals (CIs)) of reconstructed surfaces using the reduced component set were calculated for each SSM. To achieve this the models reconstructed using the reduced component set were compared to the original surface meshes. To interpret the variation explained by each PC, the mean surface was compared to surfaces generated by manipulating the retained PC up to plus/minus three standard deviations. Animated heatmaps of each PC component, where colour variation indicated the relative amount of position change of the surface nodes, were also used to assist with interpretation of PCs (see Files S1, S2 and S3).

## Results

### Tibia SSM

**Figure 1:**
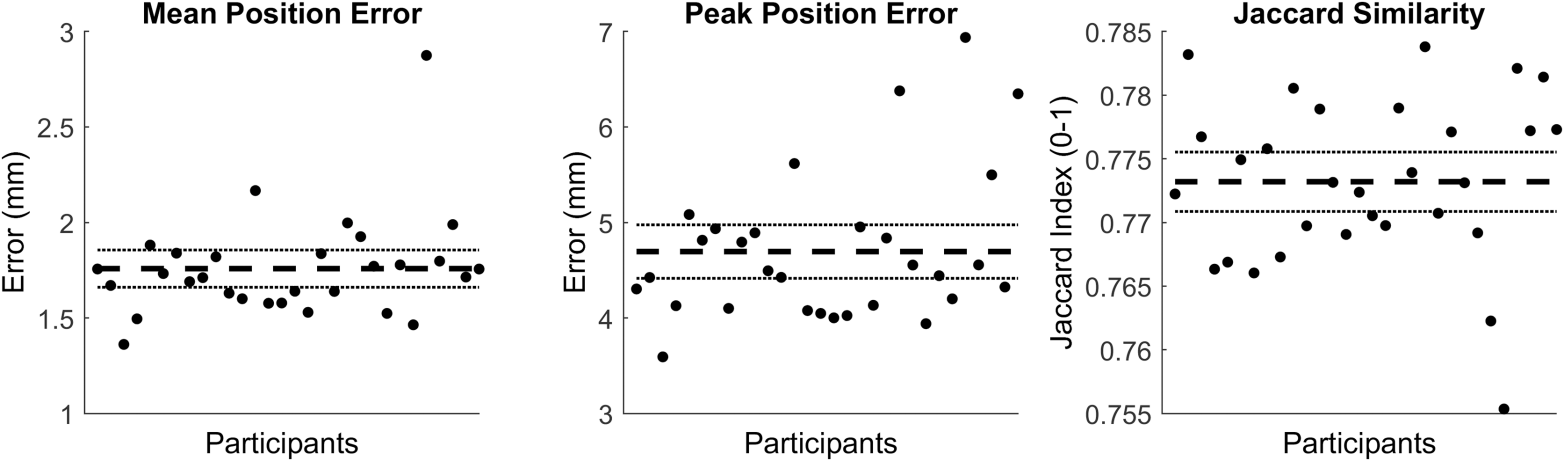
Summary of errors for the reconstructed tibia surfaces. Mean and peak error in surface node position, and the Jaccard index for the reconstructed tibia surfaces using a reduced component set. Error variables are presented in mean (dashed line) and 95% confidence intervals (dotted lines).

The first five PCs accounted for 95.80% of the geometric variation across the tibial surfaces (see Table 1). The tibia surfaces reconstructed using the reduced component set had a mean and peak error in surface node position of 1.76 mm (95% CI [1.66–1.86]) and 4.70 mm (95% CI [4.41–4.98]), they also had a Jaccard index of 0.773 (95% CI [0.771–0.776]) (see Fig. 1).

**Table 1:** Percentage of variance and interpretations of each principal component for the tibia SSM.

Principal component	% of variance	Geometric variation observed	Interpretation	
PC1	90.32	General size variation (length and width).	Lower PC1 scores described a longer and wider tibia.	Higher PC1 scores described a shorter and thinner tibia.
PC2	2.30	Midshaft thickness.	Lower PC2 scores described thicker midshaft.	Higher PC2 scores described a thinner midshaft.
PC3	1.30	General overall thickness.	Lower PC3 scores described an overall thicker tibia.	Higher PC2 scores described an overall thinner tibia.
PC4	0.95	Prominence and size of condyle plateau and tibial tuberosity. Anterior crest prominence.	Lower PC4 scores described a decreased prominence and size of the condyles, tibial plateau, and tibial tuberosity. Lower scores also described an overall decrease in the anterior crest prominence.	Higher PC4 scores described an increase in the prominence and size of the condyles, tibial plateau, and tibial tuberosity. Higher scores also described an overall increase in the anterior crest prominence.
PC5	0.93	Axial rotation.	Lower PC5 scores described axial rotation of the tibia shaft. Lower scores described external/lateral rotation at the distal end of the bone and internal/medial rotation at the proximal end.	Higher PC5 scores described internal/medial rotation at the distal end of the tibia and external/lateral rotation at the proximal end of the tibia.

**Note:**

PC, principal component.

### Tibia-fibula SSM

**Figure 2:**
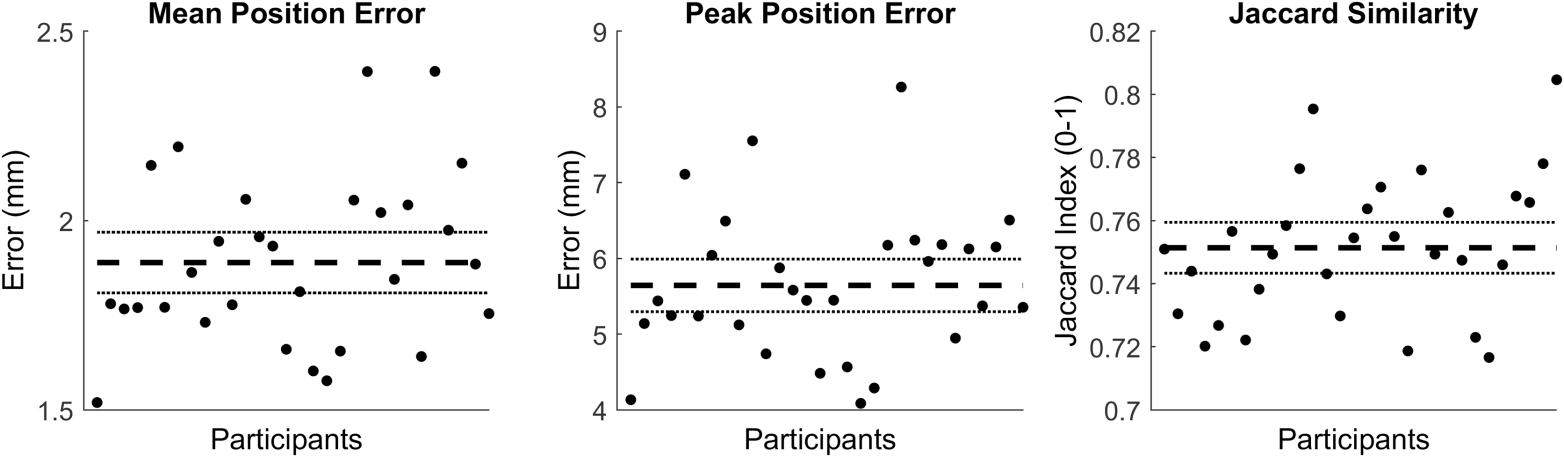
Summary of errors for the reconstructed tibia-fibula surfaces. Mean and peak error in surface node position, and the Jaccard index for the reconstructed tibia-fibula surfaces using a reduced component set. Error variables are presented in mean (dashed line) and 95% confidence intervals (dotted lines).

The first seven PCs accounted for 95.49% of the geometric variation across the tibial surfaces (see Table 2). The tibia-fibula surfaces reconstructed using the reduced component set had a mean and peak error in surface node position of 1.89 (95% CI [1.81–1.97]) and 5.67 (95% CI [5.30–5.99]), they also had a Jaccard index of 0.751 (95% CI [0.743–0.759]) (see Fig. 2).

**Table 2:** Percentage of variance and interpretations of each principal component for the tibia-fibula SSM.

Principal component	% of variance	Geometric variation observed	Interpretation	
PC1	84.24	General size variation (length and width) of tibia and fibula.	Lower PC1 scores described a longer and wider tibia and fibula	Higher PC1 scores described a shorter and thinner tibia and fibula.
PC2	3.50	Midshaft thickness of fibula. Fibula head position.	Lower PC2 scores described a thinner fibula midshaft, with a more anterior head relative to the tibia.	Higher PC2 scores described a thicker fibula midshaft, with a more posterior head relative to the tibia.
PC3	2.60	General overall thickness of tibia and fibula.	Lower PC3 scores described an overall thicker tibia and fibula,	Higher PC3 scores described an overall thinner tibia and fibula.
PC4	1.91	Tibia and Fibula anterior-posterior curvature.	Lower PC4 scores described posterior curvature of the fibula at the proximal end, the fibula head becomes more anterior. Lower scores also describe a straighter tibia.	Higher PC3 scores describe a straighter fibula, with a more posterior head. The tibia displays anterior curvature.
PC5	1.36	Fibula posterior curvature. Tibia plateau rotation.	Lower PC5 scores describe a straighter fibula, flattening in an anterior direction. Lower scores also describe internal rotation of the tibial plateau.	Higher PC5 scores describe a more posteriorly curved fibula, and a more externally rotated tibial plateau.
PC6	1.03	Tibia upper to midshaft thickness. Tibia condyle prominence.	Lower PC6 scores describe a thinner tibia, with a decreased condyle prominence.	Higher PC6 scores describe a thicker tibia with increased condyle prominence.
PC7	0.85	Interosseous width.	Lower PC7 scores describe a smaller/thinner gap between the tibia and fibula at the distal end.	Higher PC7 scores describes a bigger/thicker gap between the tibia and fibula at the distal end.

**Note:**

PC, principal component.

### Cortical-trabecular SSM

**Figure 3:**
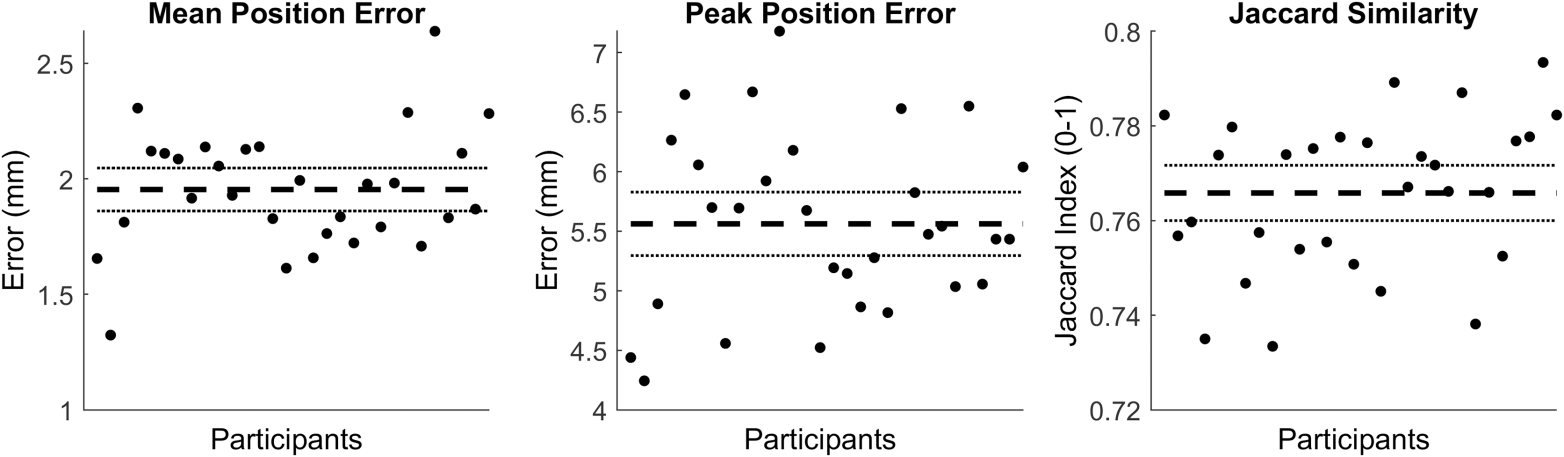
Summary of errors for the reconstructed cortical-trabecular surfaces. The mean and peak error in surface node position, and the Jaccard index for the reconstructed cortical-trabecular surfaces using a reduced component set. Error variables are presented in mean (dashed line) and 95% confidence intervals (dotted lines).

The first four PCs accounted for 95.25% of the geometric variation across the tibia trabecular (see Table 3). The tibia cortical-trabecular surfaces reconstructed using the reduced component set had a mean and peak error in surface node position of 1.95 (95% CI [1.86–2.05]) and 5.56 (95% CI [5.3–5.83]), they also had a Jaccard index of 0.766 (95% CI [0.76–0.772]) (see Fig. 3).

**Table 3:** Percentage of variance and interpretations of each principal component for the trabecular SSM.

Principal component	% of variance	Geometric variation observed	Interpretation	
PC1	87.34	General size variation (length and width).	Lower PC1 scores describes longer and wider cortical and trabecular bone.	Higher PC1 scores describes shorter and thinner cortical and trabecular bone.
PC2	4.46	Medulla cavity diameter and cortical thickness.	Lower PC2 scores describe an increased diameter of the medulla cavity, with a decrease in cortical thickness.	Higher PC2 scores describe a decrease diameter of the medulla cavity, with an increase in cortical thickness.
PC3	1.46	Anterior-posterior shaft curvature of the cortical and trabecular bone.	Lower PC3 scores describe increased prominence of the tuberosity with increased posterior curvature of both the cortical and trabecular bone.	Higher PC3 scores describe increased anterior curvature of both the cortical and trabecular bone.
PC4	1.02	Cortical bone thickness and trabecular volume at the proximal and distal ends of the tibia.	Lower PC4 scores describe a decrease in cortical thickness and an increased trabecular bone volume at the ends of the tibia.	Higher PC4 scores describe an increase in cortical thickness and a decreased trabecular bone volume at the ends of the tibia.
PC5	0.79	General overall cortical thickness.	Lower PC5 scores describe an overall decrease in cortical bone thickness.	Higher PC5 scores describe an overall increase in cortical bone thickness.

**Note:**

PC, principal component.

## Discussion

The purpose of this study was to explore tibial geometry using a SSM and provide the SSM and associated code as an open-source dataset. The data presented and associated code can be found online at https://simtk.org/projects/ssm_tibia. Our results indicated that overall size variation (length and width) was the main source of variation in all three models (*i.e*., tibia, tibia and fibula, and cortical-trabecular) accounting for 90.31%, 84.24% and 87.34%, respectively. This aligns with previous SSM of the tibia where overall size was the main source of variation in tibia (Quintens et al., 2019) and tibia-fibula models (Bruce et al., 2021). Other sources of geometric variation in our tibia surface models included overall and midshaft thickness; prominence and size of the condyle plateau, tibial tuberosity and anterior crest; and axial torsion of the tibial shaft. Sources of variation noted in the tibia model were also evident in the tibia-fibula model; however, inclusion of the fibula introduced variations in midshaft thickness of the fibula; fibula head position relative to the tibia; tibia and fibula anterior-posterior curvature; fibula posterior curvature; tibia plateau rotation; and interosseous width. However, these variations only accounted for a small proportion of overall tibia-fibula geometric variation. Lastly, sources of variation in the tibia cortical-trabecular model included variation in the medulla cavity diameter; cortical thickness; anterior-posterior shaft curvature of both the cortical and trabecular; and the volume of trabecular bone in the proximal and distal ends of the tibia. Previous research has identified both similar and different variations in tibial geometry compared to the outcomes from our research (Tümer et al., 2019; Quintens et al., 2019). Similar to the variations identified in our tibia model, past studies identified variations in the ‘bow’ of the tibia in the anterior posterior direction (*i.e*., anterior posterior shaft curvature) (Quintens et al., 2019), as well as changes in the orientation of the tibial plateau (Quintens et al., 2019). Variation in the prominence of the condyles as, to the tibial tuberosity and overall thickness of the tibia were also evident in past isolated studies of the tibia (Tümer et al., 2019; Quintens et al., 2019).

Several of the observed variations in tibial geometry could relate to increased risk of tibial stress injury. Smaller and thinner tibial geometry was the predominant source of variation found in our SSM, which can impact the bone’s ability to withstand bending forces created during exercise (Nordin & Frankel, 2012). The impact that a smaller tibia has on tibial stress injuries has been cited in both athletic (Crossley et al., 1999; Beck et al., 2014) and military populations (Giladi et al., 1991; Beck et al., 2000). However, size of bone is often proportionate to individuals’ height and mass (Duyar & Pelin, 2003). Linear regression showed that for this population that combined height and weight explained 64.1%, 63.1% and 63.9% of variance in size for the tibia, tibia-fibula, and cortical-trabecular models, respectively. Taking this into account there is approximately a 63–64% chance that the smaller tibias we observed were related to height and mass and this may not automatically be indicative of elevated tibial stress injury risk. Despite having smaller tibias, shorter individuals would likely produce reduced forces and moments during running given their lighter mass and bone segment length. Nonetheless, our shape model demonstrates that tibial size, a commonly cited risk factor for tibial stress injuries (Giladi et al., 1991; Crossley et al., 1999; Beck et al., 2000, 2014), is a primary source of variation in tibial geometry. What is likely problematic is when an individual with greater height/mass demonstrates these smaller tibial properties. Reducing tibial forces and moments during running in individuals displaying this anatomical characteristic is likely beneficial for minimising tibial stress injury risk.

PC2 of the cortical-trabecular model described the diameter of the medulla cavity, with changes to the thickness of the cortical bone. PC4 and PC5 also described changes in cortical thickness across the bone. Cortical bone thickness is cited as a risk factor for tibial stress injuries in military (Cosman et al., 2013) and athletic populations (Beck et al., 2014). Similar to smaller and thinner tibial geometries, decreased cortical thickness could impact a bone’s ability to withstand loads. Reduced thickness of the cortical bone could reduce the overall stiffness of the tibia and make it more susceptible to compression and bending forces during exercise (Nordin & Frankel, 2012). PC3 of both the tibia and tibia-fibula model described changes in overall thickness of the tibia, while PC2 of the tibia model described changes in midshaft thickness. General tibia thickness may be an important factor for tibial stress injuries (Giladi et al., 1991). However, tibial midshaft thickness is likely a more important variation than overall tibia thickness for tibial stress injury risk. Tibial stress injuries most commonly occur in the mid to distal third of the tibial shaft (Coady & Micheli, 1997; Beck, 1998). A past study found that when adjusting for body size, thinner mid-diaphysis of the tibia is an important factor in the incidence of tibial stress injuries (Popp et al., 2019). Decreased overall tibial thickness and thickness of the midshaft likely decrease the tibias’ ability to withstand bending forces created during exercise, potentially increasing the risk of tibial stress injury. Promoting running technique that minimizes tibial forces and moments in individuals with these geometries could likely be important for reducing tibial stress injury risk.

Additional areas of geometric variation we observed in the tibia, such as axial torsion of the tibia, interosseous width, and anterior-posterior curvature have not been considered in relation to tibial stress injury risk. Further, we have limited understanding on how changes in fibula geometry influence tibial stress. Previous research has identified that the fibula potentially acts in a bracing fashion, by restricting medial and posterior motion of the tibial plateau relative to the malleoli (Haider, Baggaley & Edwards, 2020). It is unknown if changes in fibula geometry might impact its ability to provide this support. Further research is warranted to better understand the effect of these additional tibial shape characteristics and fibula geometry on tibial stress, loading and injury risk.

### Practical applications of the SSM

The dataset and associated code provided at https://simtk.org/projects/ssm_tibia can be used in several ways to assist with skeletal focused research of the tibia-fibula complex. This article does not have the scope to highlight each and every potential application; however we propose three broad applications of where and how the SSM may be used. The code and analyses described in the following case studies are also included with our dataset.

### Case one: generating surface samples

**Figure 4:**
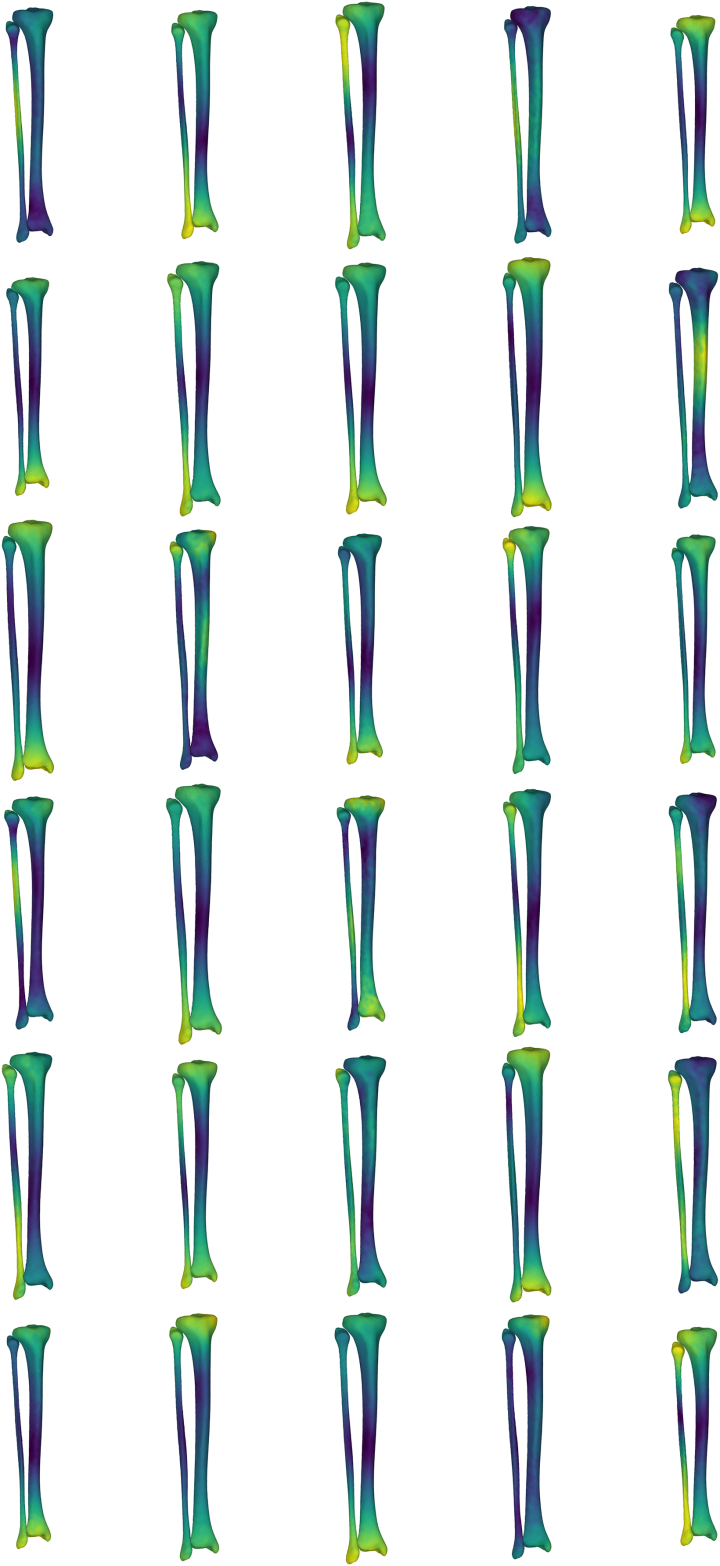
Simulated surface sample of 30 tibia-fibulas. This sample was created using the first five and the seventh principal components, opting to ignore the sixth. Standard deviation bounds were set at 1.5. Each surface is displayed with a corresponding heatmap, detailing the changes that occurred with each sample. Warmer colours describe greater change occurring at that area of tibia-fibula, respective to the mean shape model.

Obtaining CT and/or MRI scans to develop a large sample of bone surfaces can be costly from both a time and financial perspective. Further, the imaging facilities to obtain these data may not be available to all. In this case study, we provide an application that uses the SSM of the tibia-fibula to create a sample set of tibia-fibula surfaces representing standardized variation across all and/or a selection of the model components. The ‘simulatedPopulations.m’ function provided with our dataset allows users to generate a select number of surfaces from a pre-loaded shape model. While the example provided uses the tibia-fibula shape model, this process could be applied to any of the shape models included in our dataset. The function is structured to allow users to: (i) load the desired shape model; (ii) set the desired number of samples (*i.e*., *n*) to be generated; (iii) select the components of the model to be perturbed in creating the surface variations; (iv) set the magnitude of variation to perturb the model components by (in standard deviations); and (v) set the output options. The function takes these user inputs and randomly samples values for the chosen components within the standard deviation bounds provided to reconstruct *n* samples. Users can select to output a visual representation of the surfaces with heatmaps applied to highlight the areas of variation (see Fig. 4), as well as outputting the three-dimensional surfaces in STL format.

The SSM developed in our study represent the major sources of variation in the bone surfaces, and this model can be used to produce a representative sample of surfaces or a ‘simulated population.’ The ability to perturb or manipulate specific components of the shape models also allows for samples to be generated with isolated geometric variations of interest (*e.g*., specifically isolating variation in mid-shaft tibial thickness).

### Case two: predicting and generating trabecular volumes

Finite element simulations of tibial loading typically include different parts and material properties for the cortical and trabecular volumes of the bone (Edwards et al., 2010; Haider, Baggaley & Edwards, 2020). Certain scenarios can make generating surface and volumetric meshes of the trabecular more difficult or time consuming. Segmenting the trabecular surface from CT scans is possible, yet more time-consuming given the need for greater manual corrections. MRIs that are not optimized to detect the trabecular volume can also generate difficulties in segmenting the trabecular area due to lacking clearly identifiable borders between the cortical and trabecular areas. Other bone surface reconstruction methods (*e.g*., MAP-client (Zhang et al., 2014b)) only provide estimates of external bone surface and not internal trabecular volumes. Given that performing finite element simulations with both trabecular and cortical bone is more reflective of real-world scenarios, there are practical benefits to developing methods that can predict and generate trabecular volumes based on the more easily obtainable outer bone surface.

In this case study, we use the cortical-trabecular SSM to develop and evaluate a method for estimating a correspondent trabecular surface to an isolated cortical tibial surface. The theoretical hypothesis for this approach is that the outer shape of the tibia, combined with our cortical-trabecular SSM can be effective in predicting the internal trabecular shape. The ‘generateTrabecularFromSurface.m’ function provided with our dataset demonstrates the development and evaluation of this method on the dataset used in the present study, followed by the application of the method to a new tibial surface. Briefly, this code uses an optimisation procedure to predict the principal component scores for the cortical-trabecular SSM that will minimise the point-to-point error between the original outer tibia surface to that of the SSM reconstruction. The predicted scores can then be combined with the cortical-trabecular SSM to reconstruct and extract the trabecular surface. In developing this method we noted that it was common for the reconstructed trabecular surface to project outside of the tibial surface (*i.e*., not fitting within the outer tibia boundary). To combat this, we included a final step where points on the reconstructed trabecular surface that protruded outside the tibia surface were translated to the closest point one millimetre inside the boundary of the tibia.

**Figure 5:**
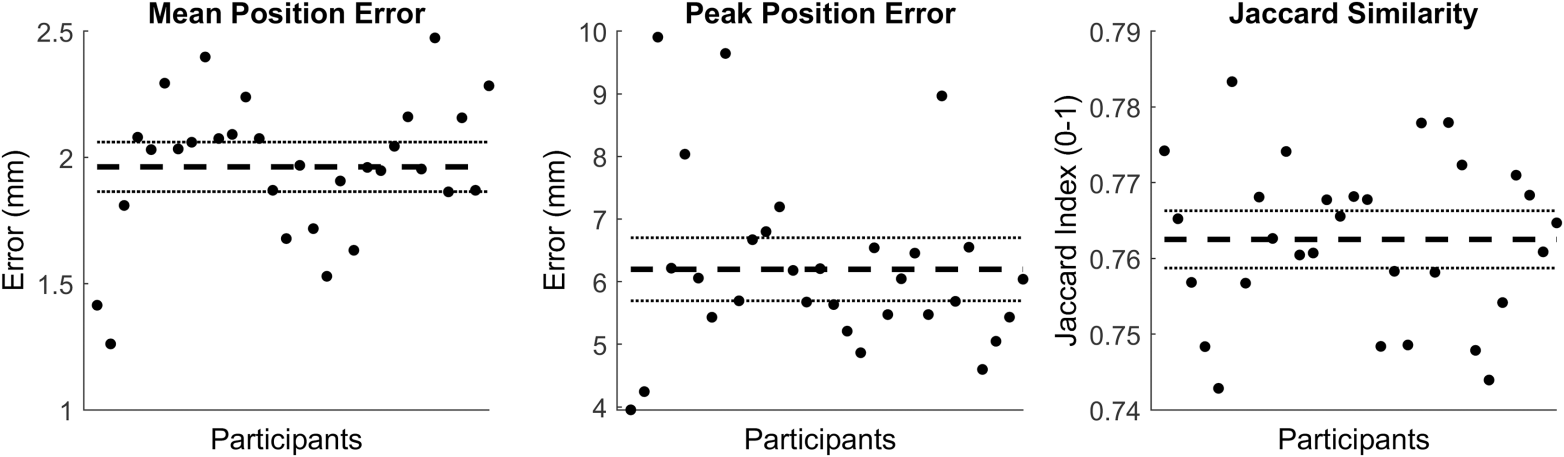
Summary of errors for the reconstructed trabecular surfaces using the prediction model. Mean and peak error in surface node position, and the Jaccard index for the reconstructed trabecular surfaces using the prediction model. Error variables are presented in mean (dashed line) and 95% confidence intervals (dotted lines).

**Figure 6:**
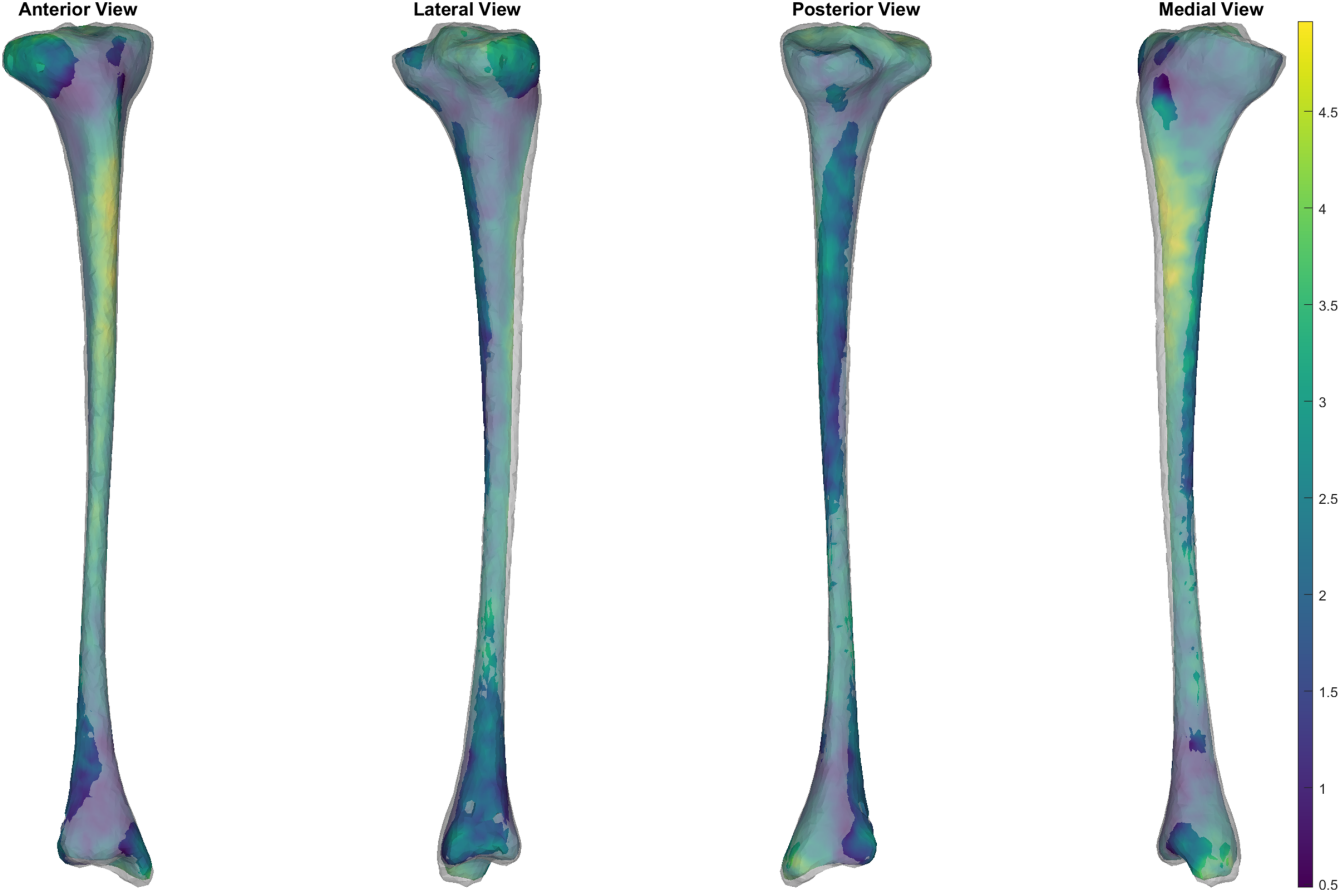
Comparison of trabecular surface reconstruction compared to the original segmentation for this participant. Comparison of the ‘average’ reconstruction (*i.e*., surface with Jaccard index closest to the mean) of the trabecular surface compared to the originally segmented trabecular for this participant. The transparent grey surface is the original segmentation and the colour map is the predicted trabecular surface. Warmer colours represent greater point distance (mm) from the original segmentation.

We evaluated the accuracy of the trabecular reconstruction method on the surfaces used to create the SSM. Specifically, we predicted the trabecular surface from only the outer cortical tibial surface and cortical-trabecular SSM using the method outlined above—and the reconstruction accuracy evaluated using the mean and peak error in surface node position, alongside the Jaccard index. Mean and peak error in surface node position (in mm) of the reconstructed trabecular surfaces were 1.96 (95% CI [1.86–2.06]) and 6.20 (95% CI [5.69–6.70]), and had a Jaccard index of 0.763 (95% CI [0.759–0.766]) (see Fig. 5). Figure 6 presents an ‘average’ performing reconstruction (*i.e*., surface with Jaccard index closest to the mean) of the trabecular surface compared to the originally segmented trabecular for this participant.

**Figure 7:**
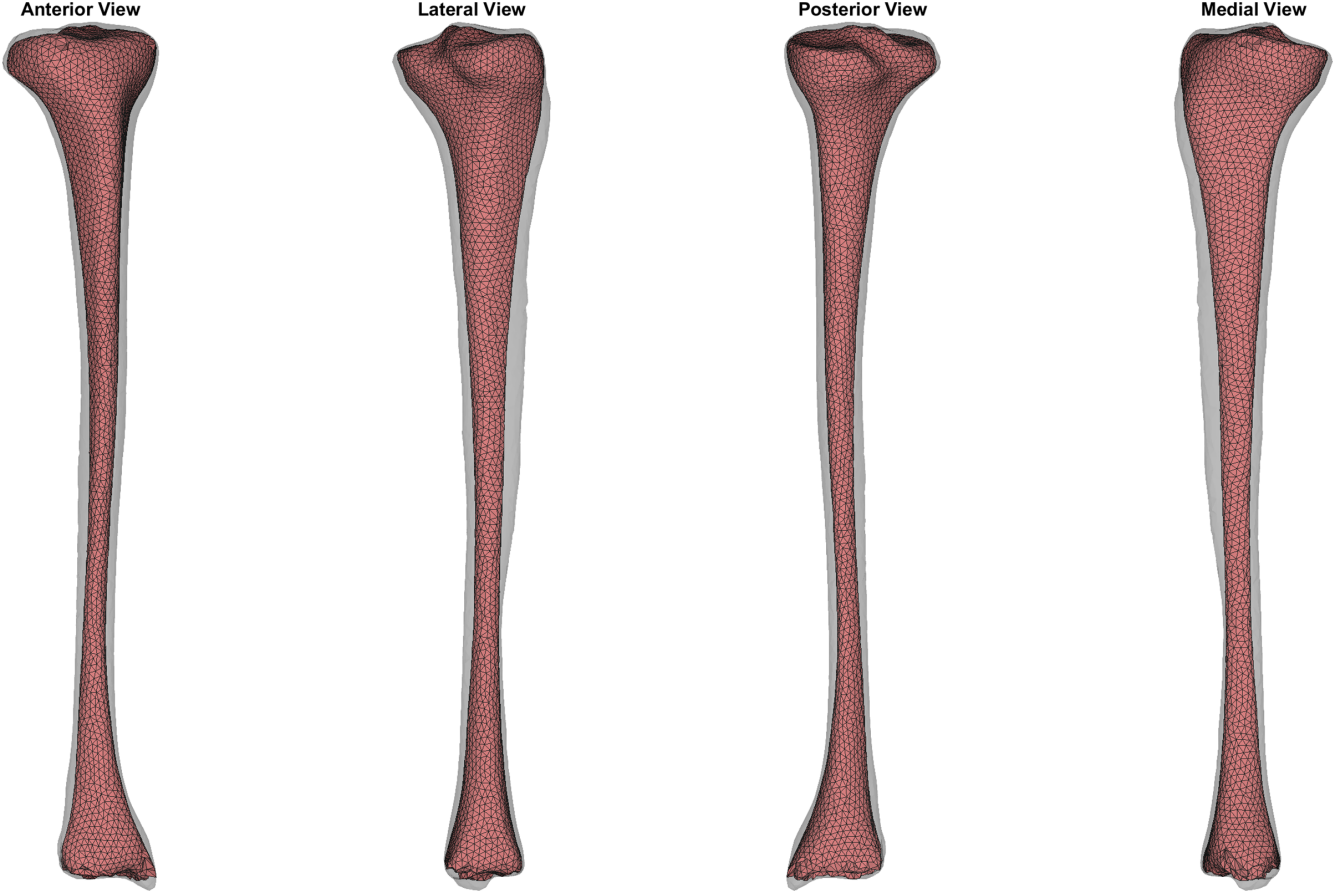
Predicted trabecular surface (red) of a randomly selected tibia (transparent grey) from Nolte et al. (2016).

This case study also provides an example of how this method can be applied to estimate trabecular volumes on a newly acquired tibial surface (Nolte et al., 2016). The trabecular of a randomly selected sample from the tibia-fibula surfaces provided by Nolte et al. (2016) was predicted using the cortical-trabecular SSM from the present study (see Fig. 7). The relative accuracy of this specific trabecular reconstruction cannot be evaluated due to Nolte et al. (2016) only providing the outer tibial surface meshes. Qualitative evaluation of the predicted trabecular surface from this new sample revealed potential quality issues towards the distal end of the bone. Nonetheless, the code is provided to demonstrate how this method can be applied to any tibial surface data with the caveat that quality control checks following predictions are likely required.

### Case three: generating tibia-fibula surfaces from palpable landmarks

Providing methods to generate three-dimensional models of the tibia-fibula allow those who cannot acquire medical images to use these models within their work or practice. Obtaining spatial measurements from palpable landmarks on the tibia-fibula surface is a much more accessible method and can be achieved through motion capture or video-based methods. Past work (Bruce et al., 2021) has demonstrated that relatively accurate reconstructions of tibia-fibula surfaces can be obtained by fitting SSM to palpable landmarks *via* optimization procedures.

In this case study, we provide a similar application to that outlined by Bruce et al. (2021). We combine our SSM of the tibia-fibula with a set of measured palpable landmarks to reconstruct the entire surfaces. The ‘generateSurfaceFromLandmarks.m’ function provided with our dataset demonstrates an optimization procedure which fits the tibia-fibula SSM to a set of palpable landmarks on a new set of tibia-fibula surfaces (Nolte et al., 2020). We used the same set of palpable landmarks as Bruce et al. (2021), those being the tibial tuberosity, medial condyle, lateral and medial malleoli, lateral aspect of the fibula head, anterior border of the tibia at 25%, 50% and 75% of the distance between the medial condyle and malleolus, and the lateral fibula diaphysis at 25% of the distance from the lateral malleolus to the lateral point on the fibula head. These landmarks were digitized on the mean surface from the tibia-fibula SSM, as well as on 35 surfaces from the new dataset (Nolte et al., 2020). We performed nodal correspondence and registration of the new surfaces to the SSM mean using the coherent drift point algorithm and Procrustes analysis, respectively. The principal component scores from the SSM were then manipulated within an optimization that minimized the summed Euclidean distance between the landmarks on the SSM mean surface and new surface.

**Figure 8:**
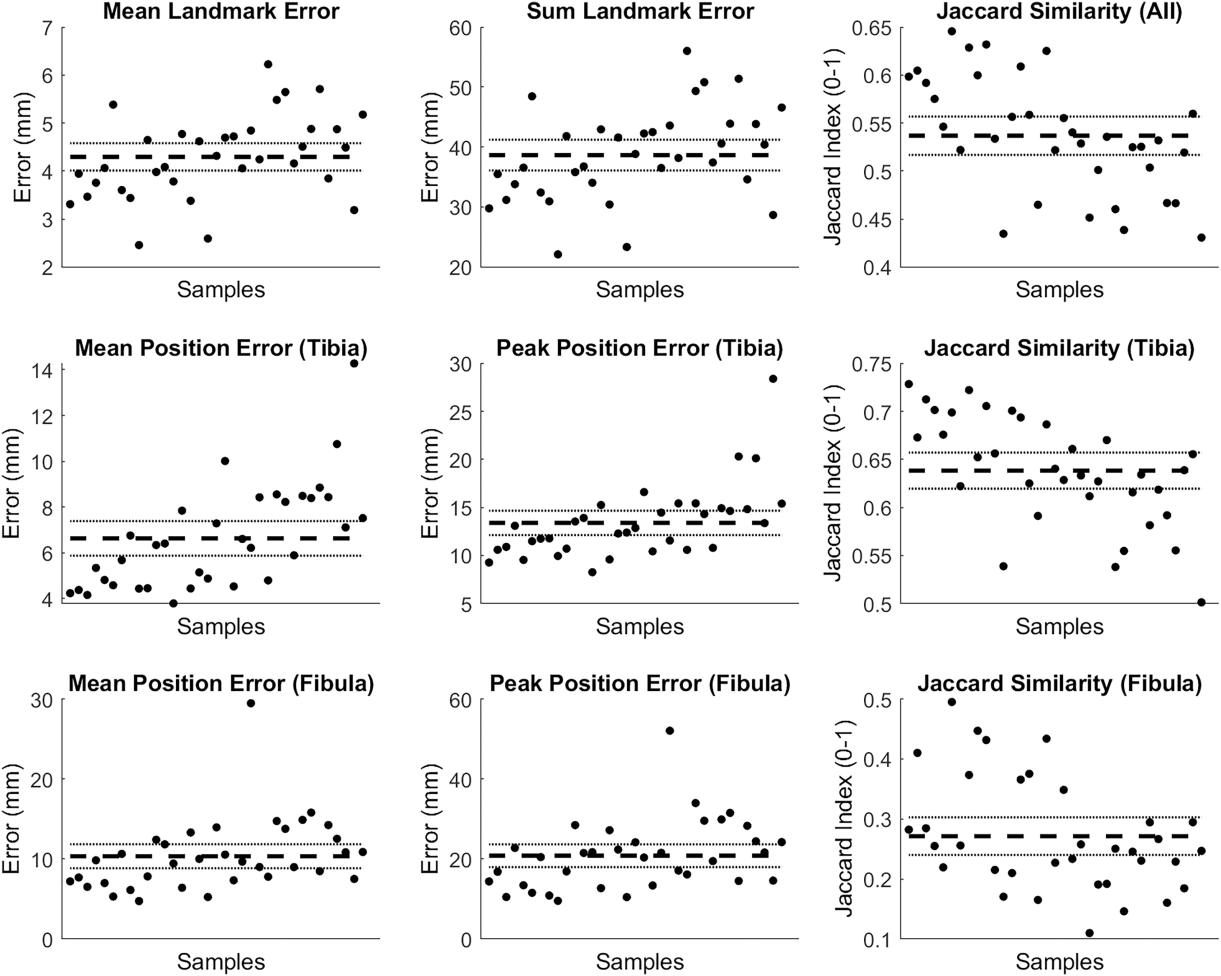
Summary of errors for the tibia and fibula reconstructed from palpable landmarks. The mean and summed error, and Jaccard index for landmark positions (row 1). Mean and peak error of the surface node position, and the Jaccard index for the tibia surfaces (row 2) and fibula surface (row 3) reconstructed using the palpable landmarks. Error variables are presented in mean (dashed line) and 95% confidence intervals (dotted lines).

The mean and summed error in landmark positions (in mm) were 4.29 (95% CI [4.01–4.58]) and 38.65 (95% CI [36.09–41.22]), respectively; while the Jaccard index values for the entire reconstruction were 0.537 (95% CI [0.517–0.557]) (see row 1 of Fig. 8). Mean and peak error in surface node position (in mm) for the tibia surface were 6.63 (95% CI [5.88–7.39]) and 13.39 (95% CI [12.12–14.65]), respectively. The Jaccard index for the reconstructed tibia surfaces was 0.638 (95% CI [0.620–0.657]) (see row 2 of Fig. 8). Mean and peak error in surface node position (in mm) for the fibula, and the Jaccard index for the reconstructed fibula surfaces were 10.34 (95% CI [8.85–11.84]), 20.82 (95% CI [17.97–23.67]) and 0.271 (95% CI [0.240–0.303]), respectively (see row 3 of Fig. 8).

**Figure 9:**
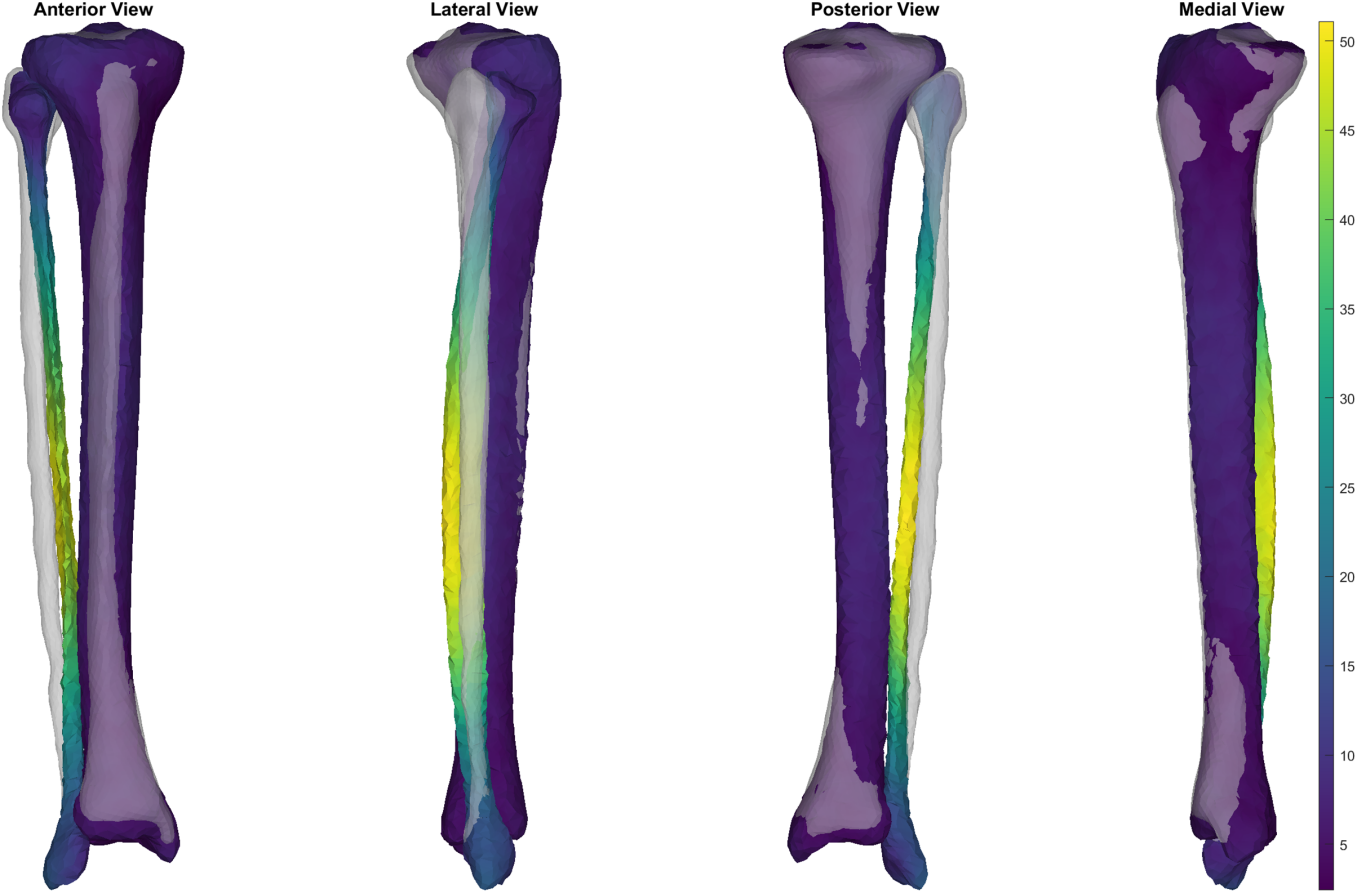
A poor reconstruction of the fibula from a minimised set of palpable landmarks. Example of a ‘poor’ reconstruction of the fibula from a minimised set of palpable landmarks. The transparent grey surface is the original segmentation, and the color map is the reconstructed surface. Warmer colours represent greater point distance (mm) from the original segmentation.

**Figure 10:**
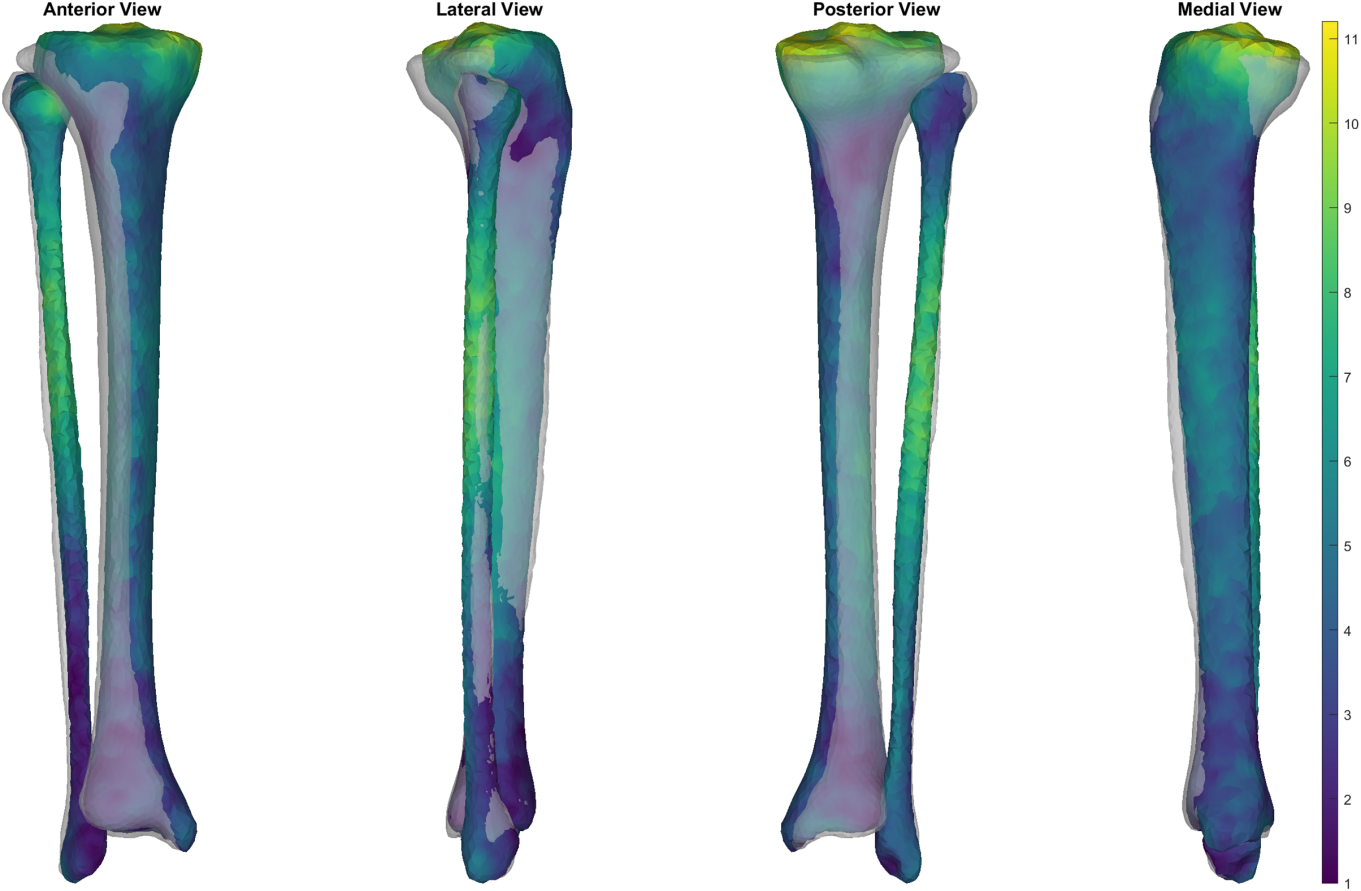
A good reconstruction of the tibia-fibula from a minimised set of palpable landmarks. Example of a ‘good’ reconstruction of the tibia and fibula from a minimised set of palpable landmarks The transparent grey surface is the original segmentation, and the color map is the reconstructed surface. Warmer colours represent greater point distance (mm) from the original segmentation.

This case study provides an example of how our statistical shape model can be used with a minimal set of palpable landmarks on the tibia and fibula to estimate a 3D surface of the bones. The tibia can be reconstructed with reasonable accuracy. However, similar to Bruce et al. (2021), we noted better reconstruction performance and accuracy for the tibia over the fibula. The greater number and spread of landmarks on the tibia (*i.e*., seven on the tibia *vs* two on the fibula), and focus of the SSM components on the tibia are the likely reasons for this. Curvature of the fibula and relative position of the fibula head were common areas where larger discrepancies occurred (see Fig. 9). Estimating additional landmarks at further points along the fibula or altering the code to give different weightings across landmarks in the optimisation may help address these issues. These may be particularly important for those who wish to use this code that have a specific interest in fibula anatomy and function. We also noted variation between samples with respect to reconstruction accuracy (see Figs. 9 and 10). The variable performance was likely due to how well the original SSM captured the shape characteristics present in the individual samples.

### Limitations

The cadavers used were predicted to be from healthy adults (using meta-data provided by NMDID). Most participants meta-data included past medical history; however, we cannot be sure of the accuracy of all details. Hence, we cannot confirm that participants had no prior illness or injury that effected bone growth and formation.

The tibia-fibula set used to create the SSM was comprised of adults between the ages of 19 and 40. This may limit the applicability of the SSM outcomes to groups outside this age range, particularly geriatric and pediatric populations. For example, Bruce et al. (2021) demonstrated that surface reconstruction accuracy decreased when their SSM was applied to older populations, particularly in those whose bone may be affected by age-related degradation. Ethnicity is also known to affect bone geometry (Mahfouz et al., 2011; Sintini et al., 2018). While some included participants were born outside the United States, no data on ethnicity was available. The SSM should therefore not be used to examine ethnic factors related to bone geometry.

Lastly, this research was only conducted using the right tibia-fibula, in order to complete analysis on the left tibia-fibula users could employ a mirroring approach to allow complete left tibia-fibula reconstructions.

## Conclusions

Across all three of our SSM the largest source of geometric variation was a general size variation, with changes in both length and width observed. Important variations that could increase the risk of tibial stress injury were observed in the tibia and tibia-fibula SSM. These included general tibial thickness, midshaft thickness, tibial length, and medulla cavity diameter (indicative of cortical thickness). Further research is warranted to better understand the effect of these tibial and fibula shape characteristics on tibial stress, loading and injury risk. This SSM and associated code has been provided in an opensource dataset for use within the research community. The associated code includes three example applications, these include generation of a random sample, reconstruction of trabecular surfaces and reconstruction from palpable landmarks. In providing this dataset we hope to improve research skills in those individuals who may not have access to the knowledge or training. Further, this dataset could help to improve understanding of bone stress injuries, and potentially assist other research such as the implementation of medical devices (*i.e*., plates and braces). The SSM and all available code will be available for use at (https://simtk.org/projects/ssm_tibia).

## Supplemental Information

10.7717/peerj.14708/supp-1 Supplemental Information 1 Animated heatmaps for the tibia model principal components.

10.7717/peerj.14708/supp-2 Supplemental Information 2 Animated heatmaps for the tibia-fibula principal components.

10.7717/peerj.14708/supp-3 Supplemental Information 3 Animated heatmaps for the cortical-trabecular prinicpal components.

## Additional Information and Declarations

### Data Availability

The following information was supplied regarding data availability: The raw data and code for this project is available at Figshare: Keast, Meghan; Bonacci, Jason; Fox, Aaron (2022): Statistical Shape Model of the Tibia.. figshare. Dataset. https://doi.org/10.6084/m9.figshare.20454462.v3. The computed tomography scans obtained from the New Mexico Decedent Image Database cannot be shared publicly as registration and approval is required from the creators. However, the public can access these through the appropriate steps: https://nmdid.unm.edu/. Individuals wishing to view the scans will need to register and request the appropriate medical imagery. The case IDs for the scans can be found in the above linked repository under the segmentation folder. When requesting the images, these can be used to acquire the correct scans.
