# Supplementary figures and images for: Geometric variation of the human tibia-fibula: a public dataset of tibia-fibula surface meshes and statistical shape model
Source: PeerJ. 2023 Feb 16;11:e14708. doi: 10.7717/peerj.14708 (PMC9939022; doi:10.7717/peerj.14708)

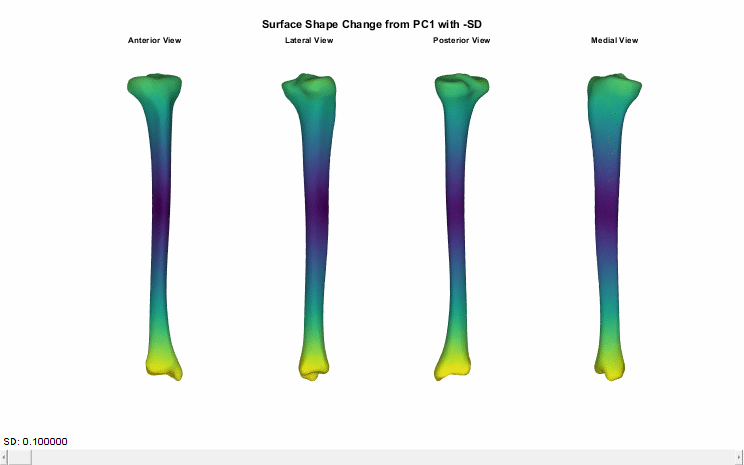

Supplement: Supplemental Information 1 [file peerj-11-14708-s001.zip › PC1_minus-3SD_animation.gif]

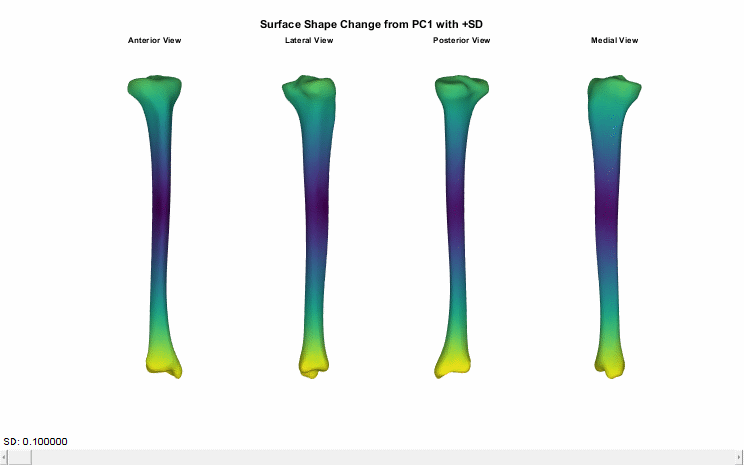

Supplement: Supplemental Information 1 [file peerj-11-14708-s001.zip › PC1_plus-3SD_animation.gif]

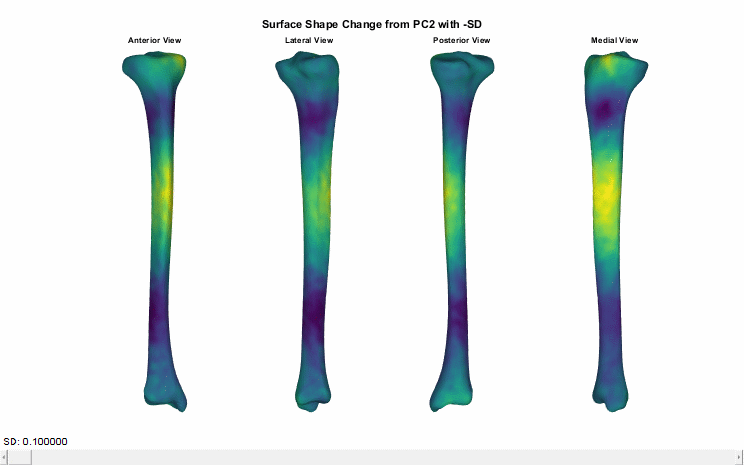

Supplement: Supplemental Information 1 [file peerj-11-14708-s001.zip › PC2_minus-3SD_animation.gif]

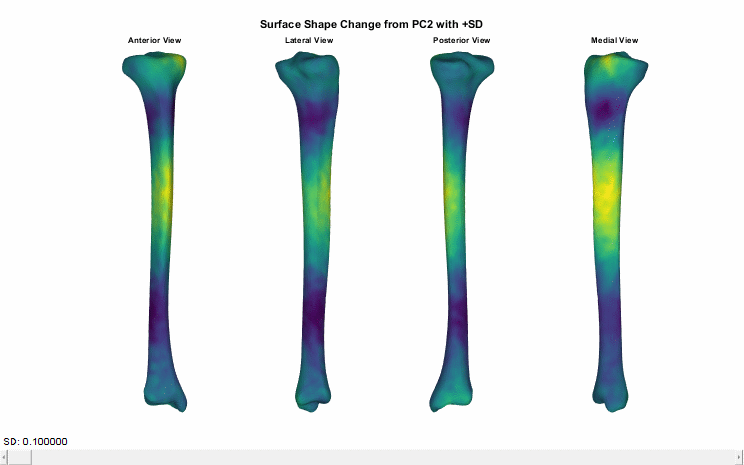

Supplement: Supplemental Information 1 [file peerj-11-14708-s001.zip › PC2_plus-3SD_animation.gif]

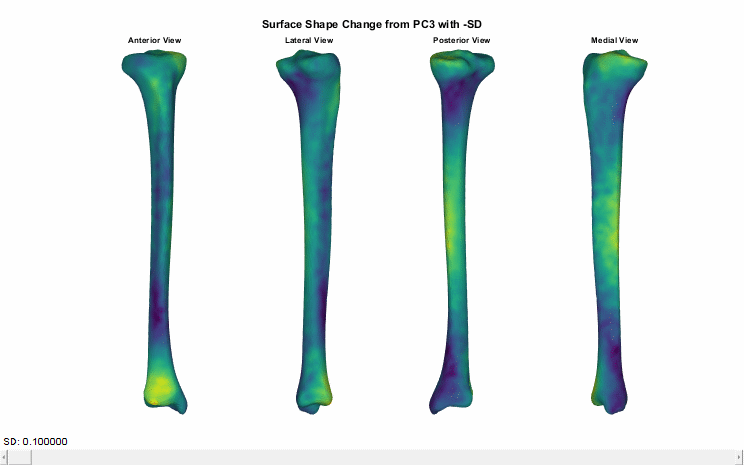

Supplement: Supplemental Information 1 [file peerj-11-14708-s001.zip › PC3_minus-3SD_animation.gif]

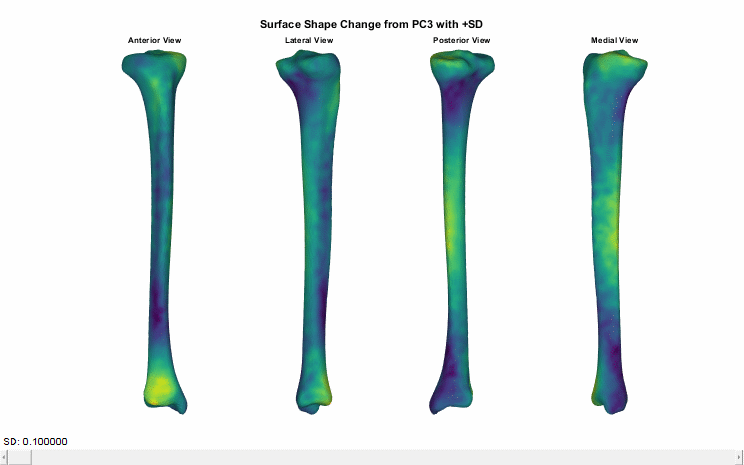

Supplement: Supplemental Information 1 [file peerj-11-14708-s001.zip › PC3_plus-3SD_animation.gif]

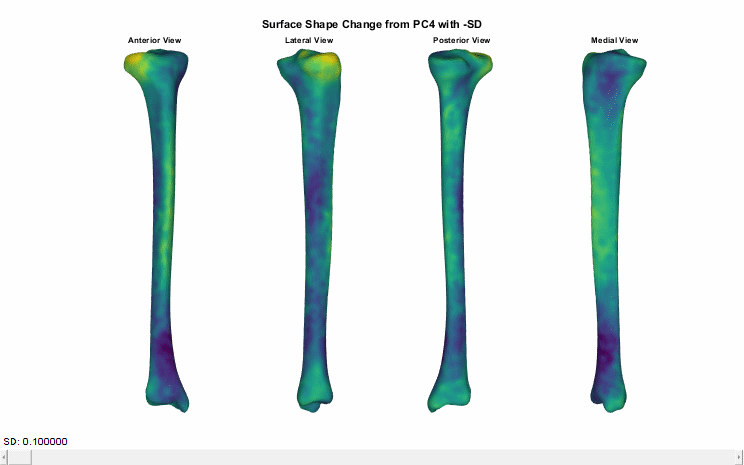

Supplement: Supplemental Information 1 [file peerj-11-14708-s001.zip › PC4_minus-3SD_animation.gif]

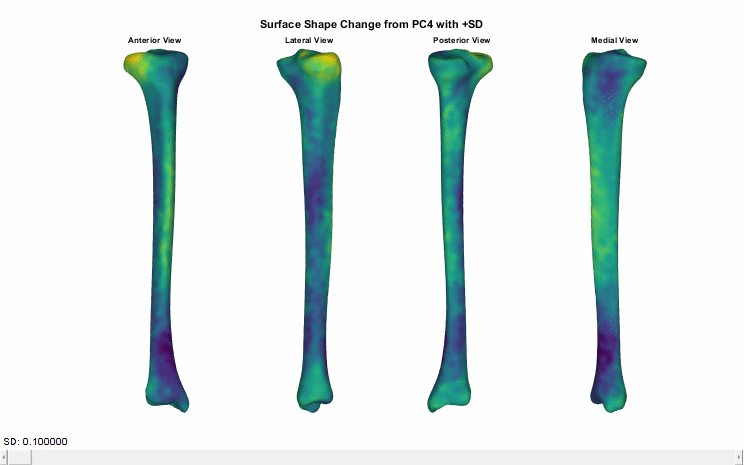

Supplement: Supplemental Information 1 [file peerj-11-14708-s001.zip › PC4_plus-3SD_animation.gif]

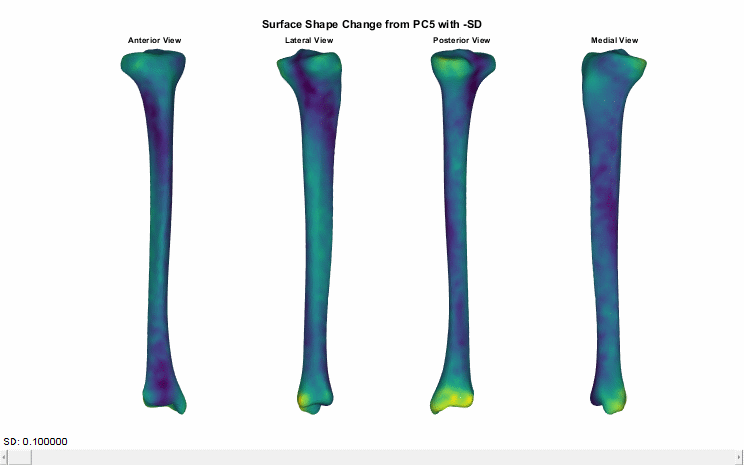

Supplement: Supplemental Information 1 [file peerj-11-14708-s001.zip › PC5_minus-3SD_animation.gif]

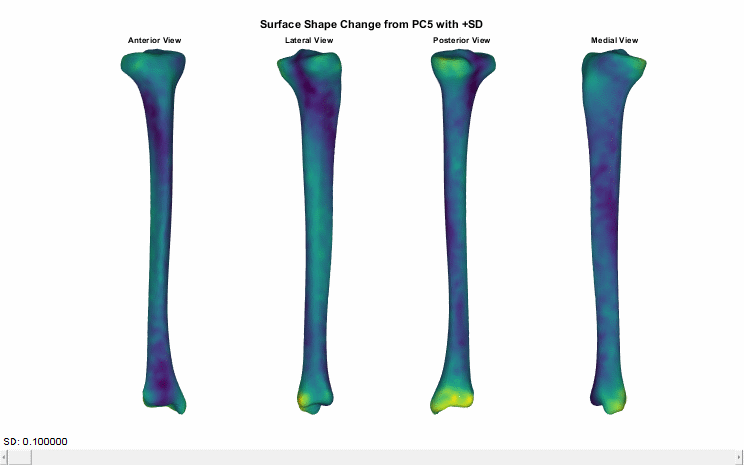

Supplement: Supplemental Information 1 [file peerj-11-14708-s001.zip › PC5_plus-3SD_animation.gif]

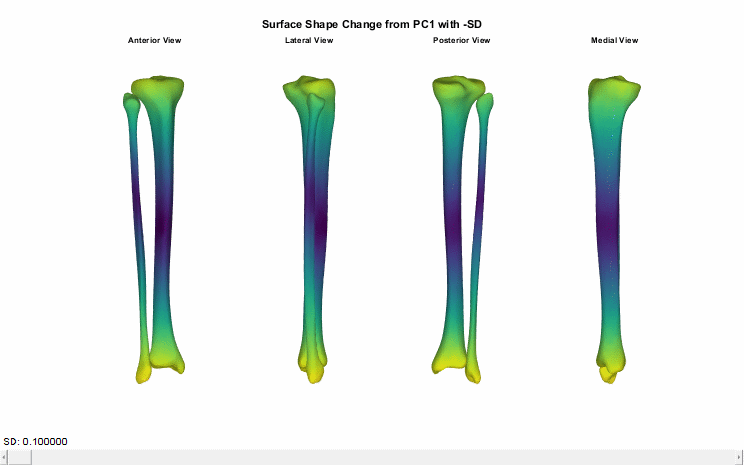

Supplement: Supplemental Information 2 [file peerj-11-14708-s002.zip › PC1_minus-3SD_animation.gif]

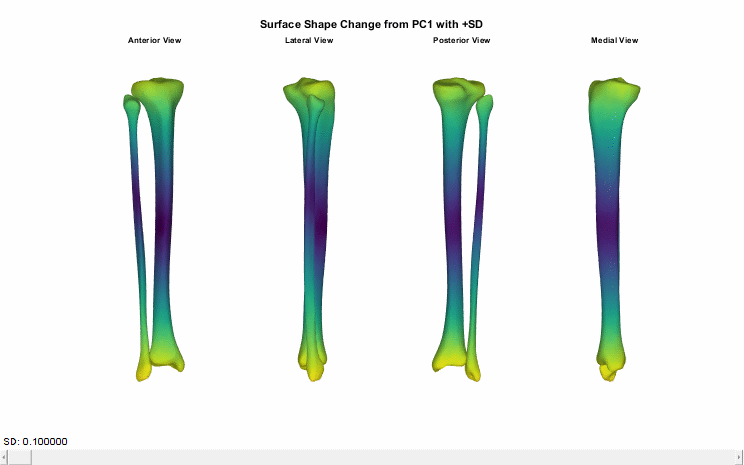

Supplement: Supplemental Information 2 [file peerj-11-14708-s002.zip › PC1_plus-3SD_animation.gif]

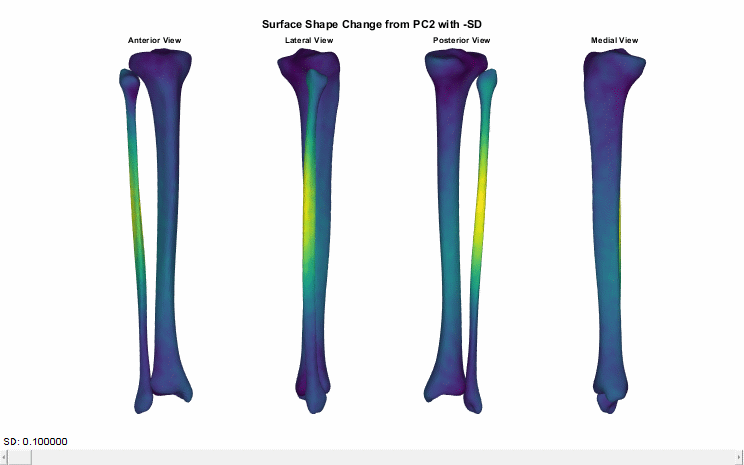

Supplement: Supplemental Information 2 [file peerj-11-14708-s002.zip › PC2_minus-3SD_animation.gif]

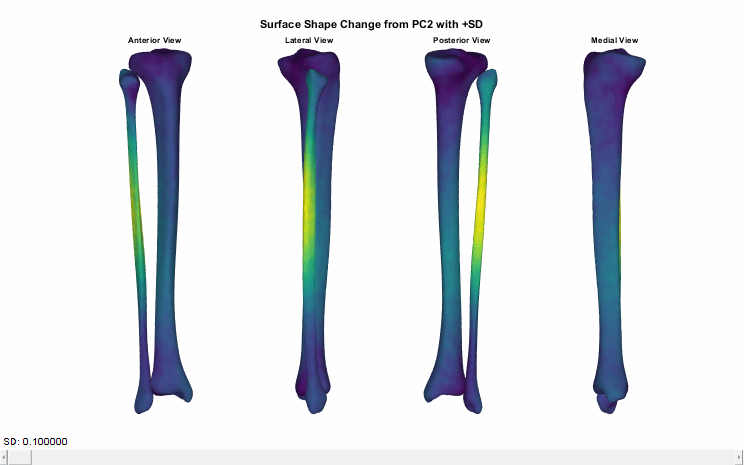

Supplement: Supplemental Information 2 [file peerj-11-14708-s002.zip › PC2_plus-3SD_animation.gif]

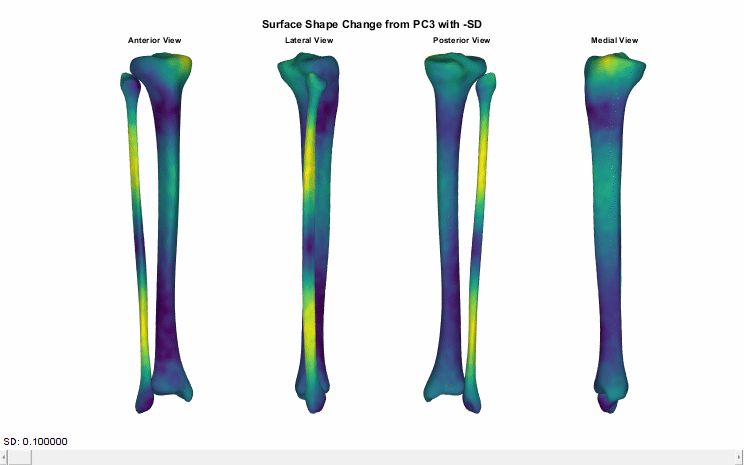

Supplement: Supplemental Information 2 [file peerj-11-14708-s002.zip › PC3_minus-3SD_animation.gif]

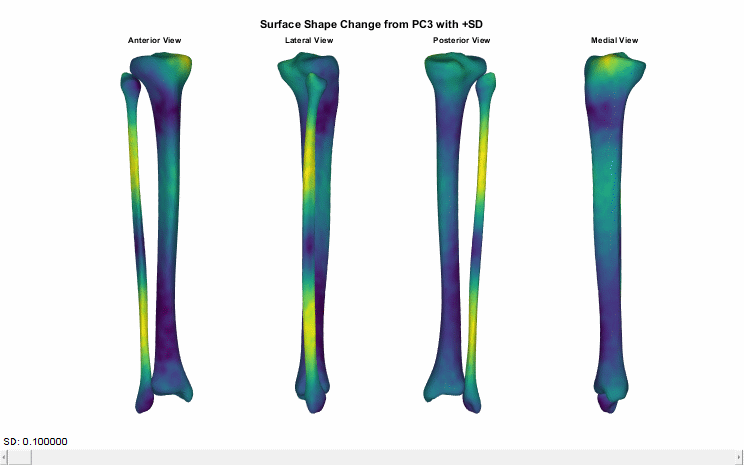

Supplement: Supplemental Information 2 [file peerj-11-14708-s002.zip › PC3_plus-3SD_animation.gif]

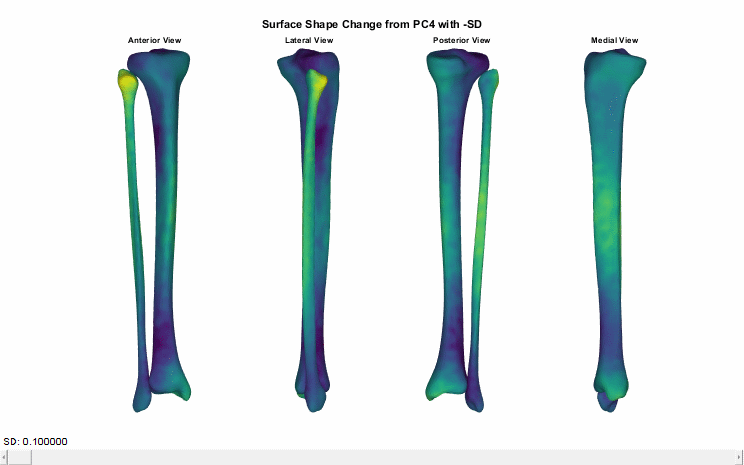

Supplement: Supplemental Information 2 [file peerj-11-14708-s002.zip › PC4_minus-3SD_animation.gif]

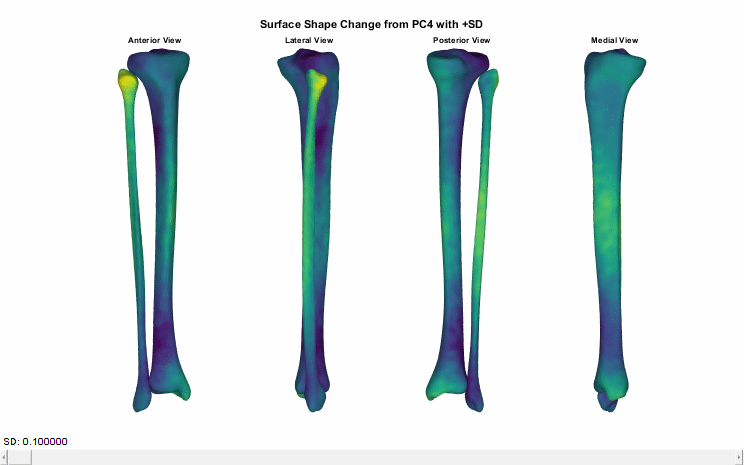

Supplement: Supplemental Information 2 [file peerj-11-14708-s002.zip › PC4_plus-3SD_animation.gif]

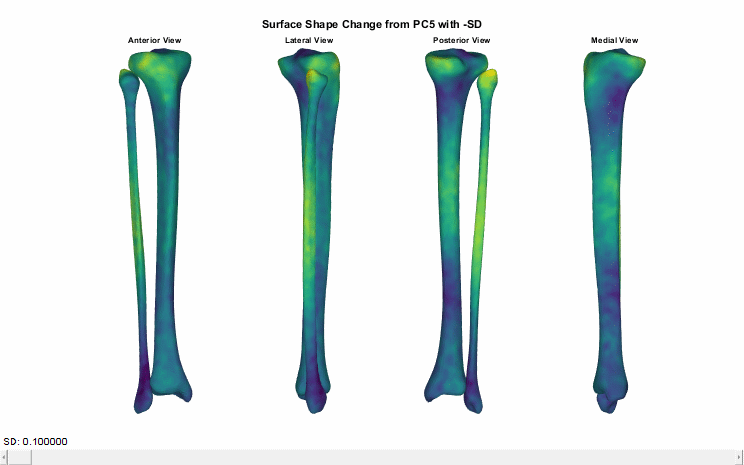

Supplement: Supplemental Information 2 [file peerj-11-14708-s002.zip › PC5_minus-3SD_animation.gif]

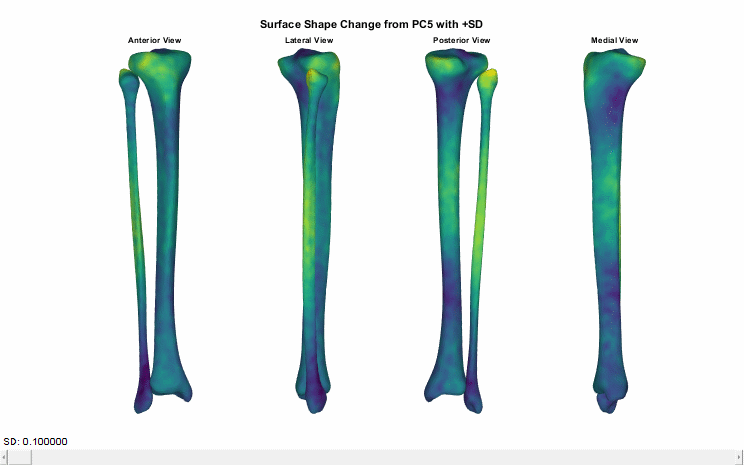

Supplement: Supplemental Information 2 [file peerj-11-14708-s002.zip › PC5_plus-3SD_animation.gif]

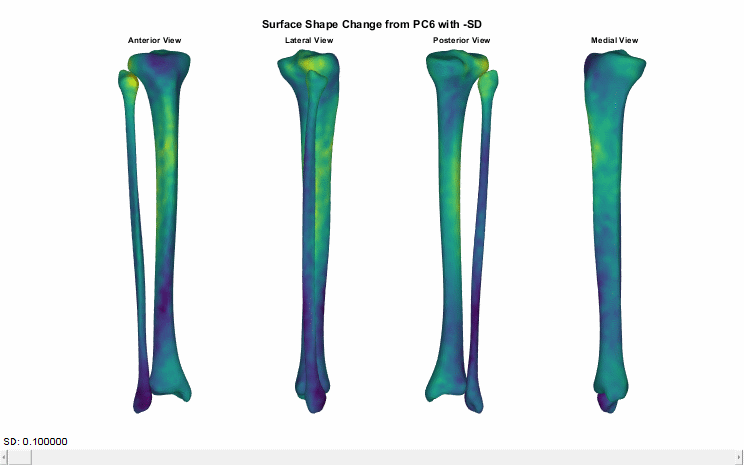

Supplement: Supplemental Information 2 [file peerj-11-14708-s002.zip › PC6_minus-3SD_animation.gif]

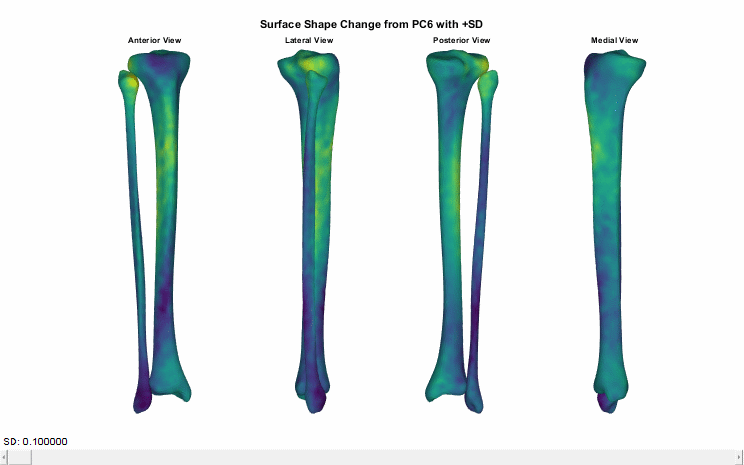

Supplement: Supplemental Information 2 [file peerj-11-14708-s002.zip › PC6_plus-3SD_animation.gif]

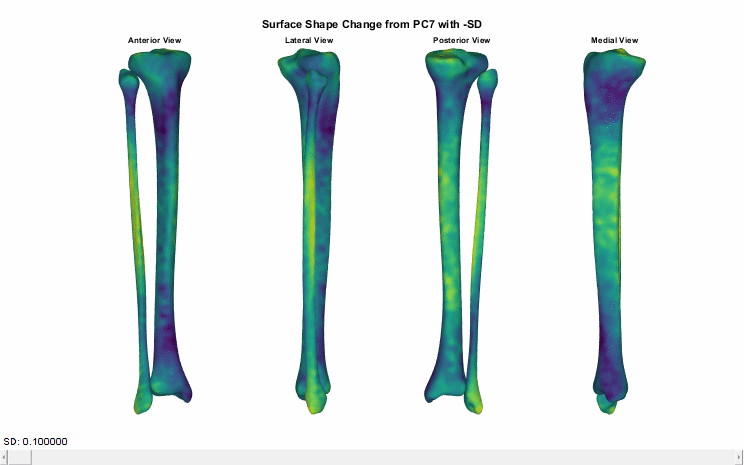

Supplement: Supplemental Information 2 [file peerj-11-14708-s002.zip › PC7_minus-3SD_animation.gif]

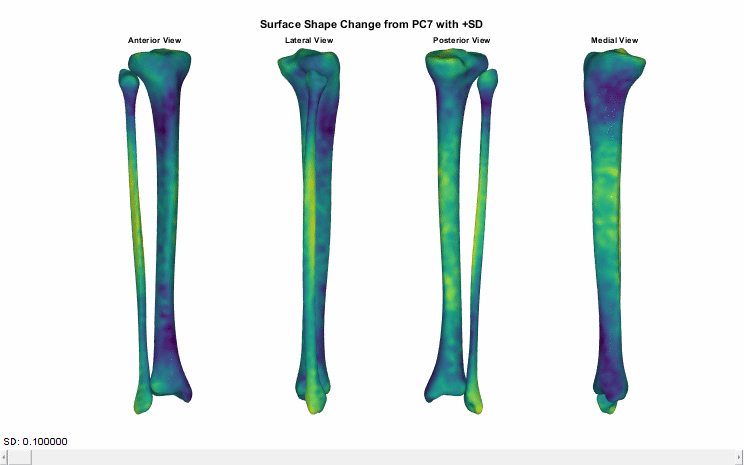

Supplement: Supplemental Information 2 [file peerj-11-14708-s002.zip › PC7_plus-3SD_animation.gif]

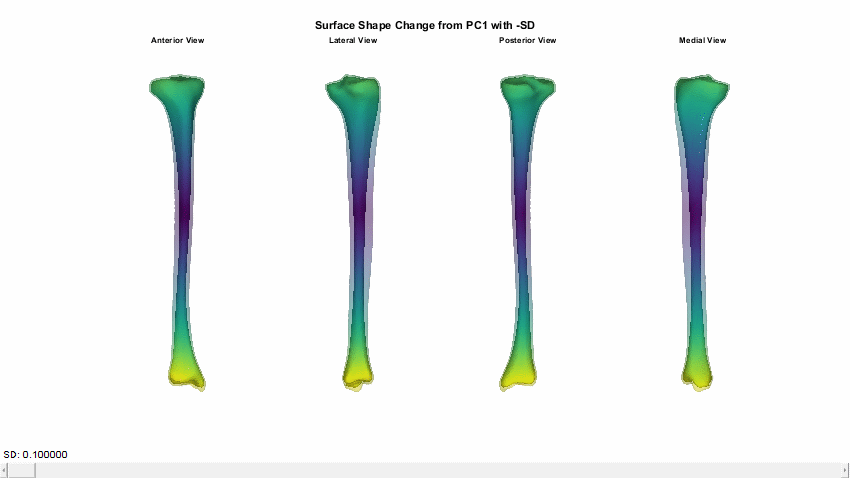

Supplement: Supplemental Information 3 [file peerj-11-14708-s003.zip › PC1_minus-3SD_animation.gif]

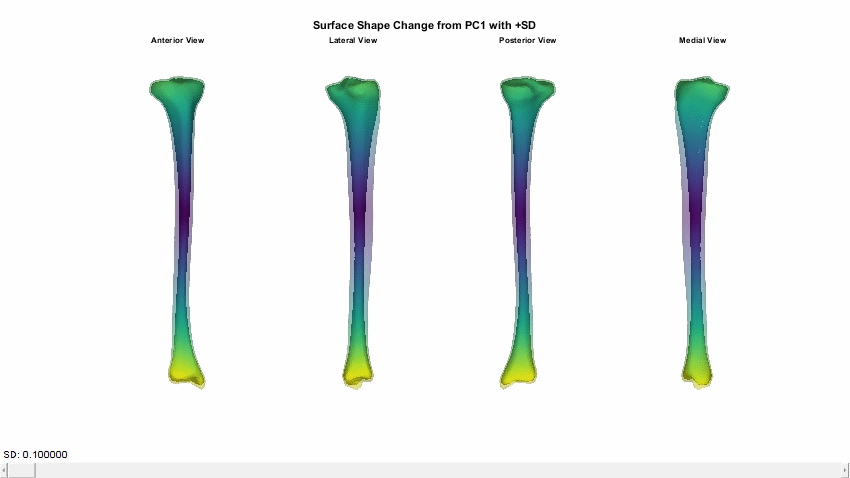

Supplement: Supplemental Information 3 [file peerj-11-14708-s003.zip › PC1_plus-3SD_animation.gif]

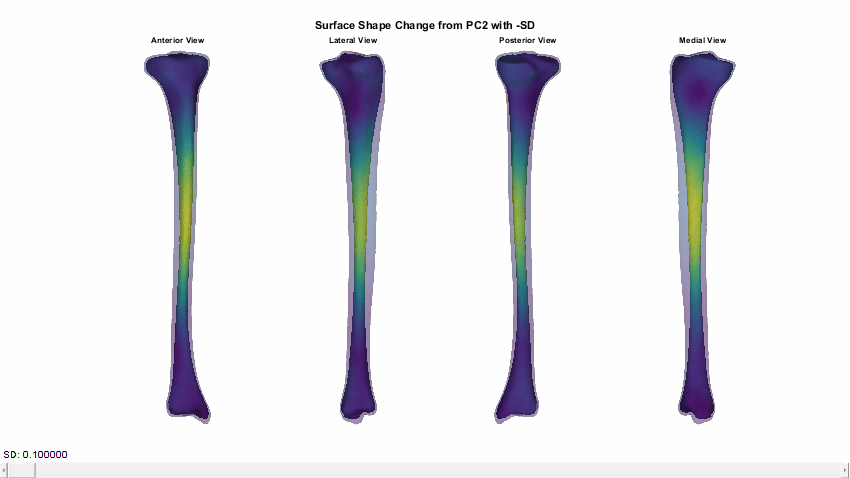

Supplement: Supplemental Information 3 [file peerj-11-14708-s003.zip › PC2_minus-3SD_animation.gif]

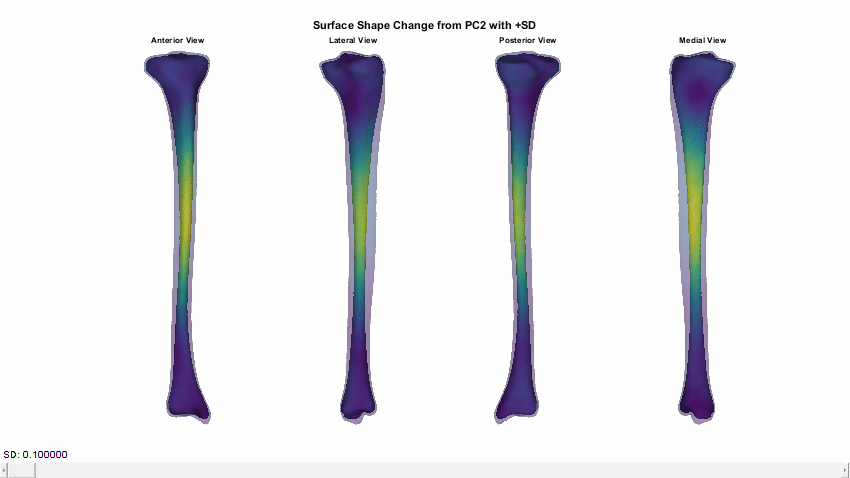

Supplement: Supplemental Information 3 [file peerj-11-14708-s003.zip › PC2_plus-3SD_animation.gif]

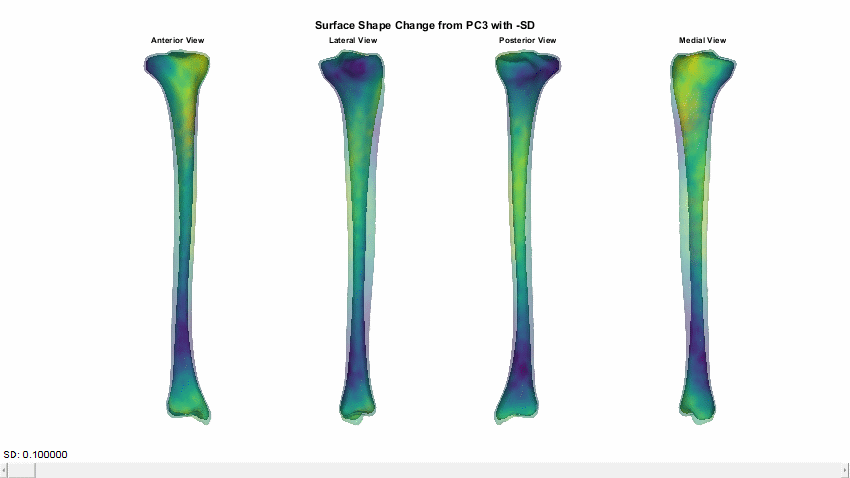

Supplement: Supplemental Information 3 [file peerj-11-14708-s003.zip › PC3_minus-3SD_animation.gif]

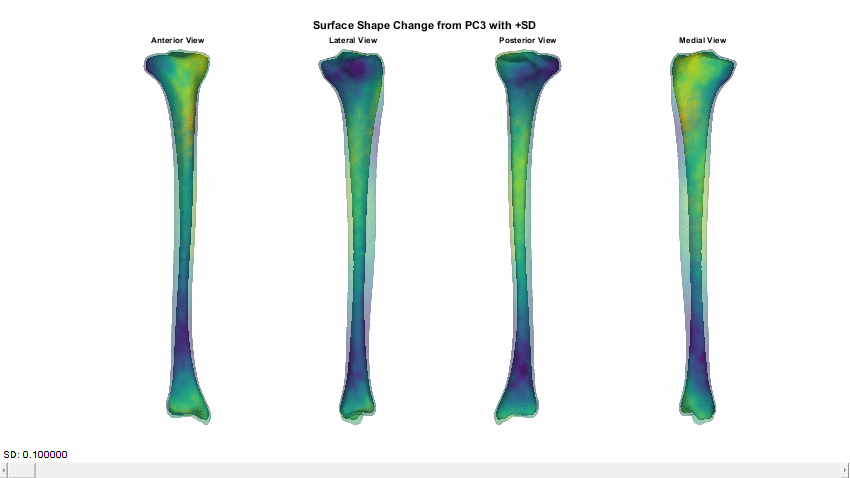

Supplement: Supplemental Information 3 [file peerj-11-14708-s003.zip › PC3_plus-3SD_animation.gif]

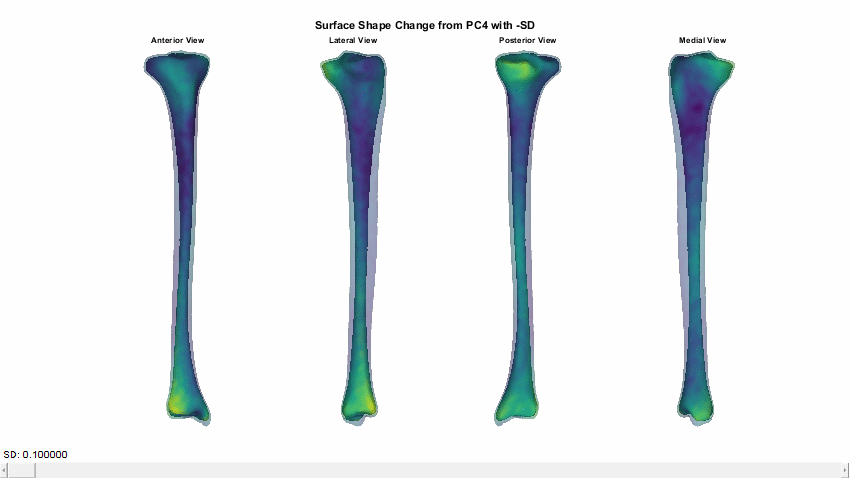

Supplement: Supplemental Information 3 [file peerj-11-14708-s003.zip › PC4_minus-3SD_animation.gif]

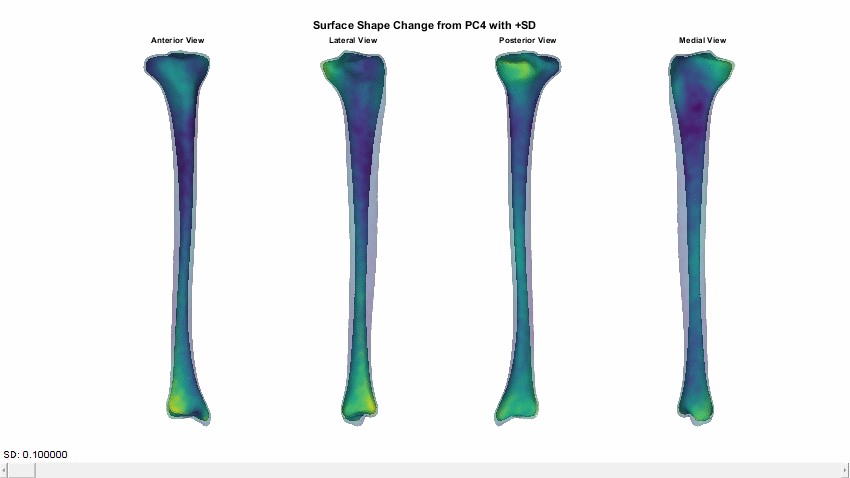

Supplement: Supplemental Information 3 [file peerj-11-14708-s003.zip › PC4_plus-3SD_animation.gif]

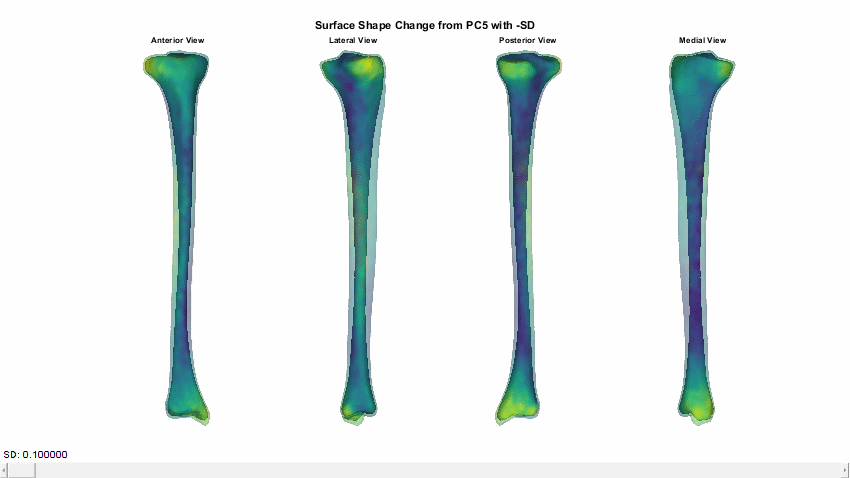

Supplement: Supplemental Information 3 [file peerj-11-14708-s003.zip › PC5_minus-3SD_animation.gif]

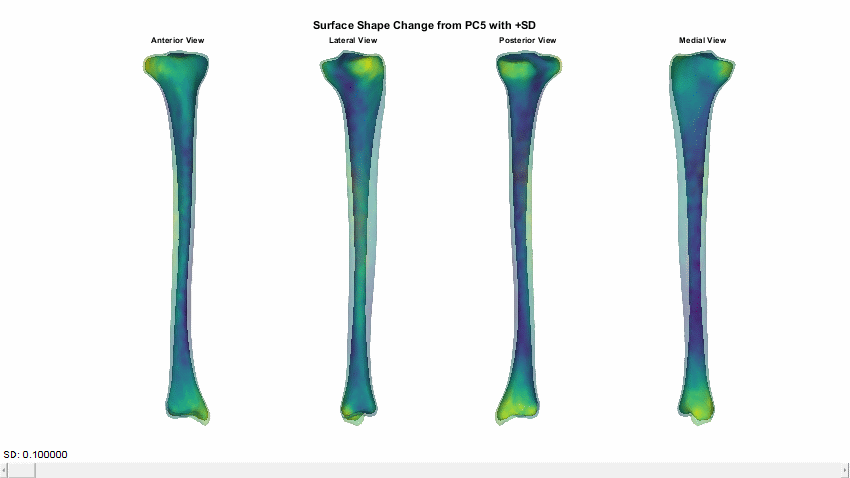

Supplement: Supplemental Information 3 [file peerj-11-14708-s003.zip › PC5_plus-3SD_animation.gif]
